# Supplementary material for: Genome sequence and analysis of the Japanese morning glory Ipomoea nil
Source: Nat Commun. 2016 Nov 8;7:13295. doi: 10.1038/ncomms13295 (PMC5105172; doi:10.1038/ncomms13295)
Supplement: Supplementary Information — Supplementary Figures 1-20, Supplementary Tables 1-24, Supplementary Methods and Supplementary References [file ncomms13295-s1.pdf]

Supplementary Figures

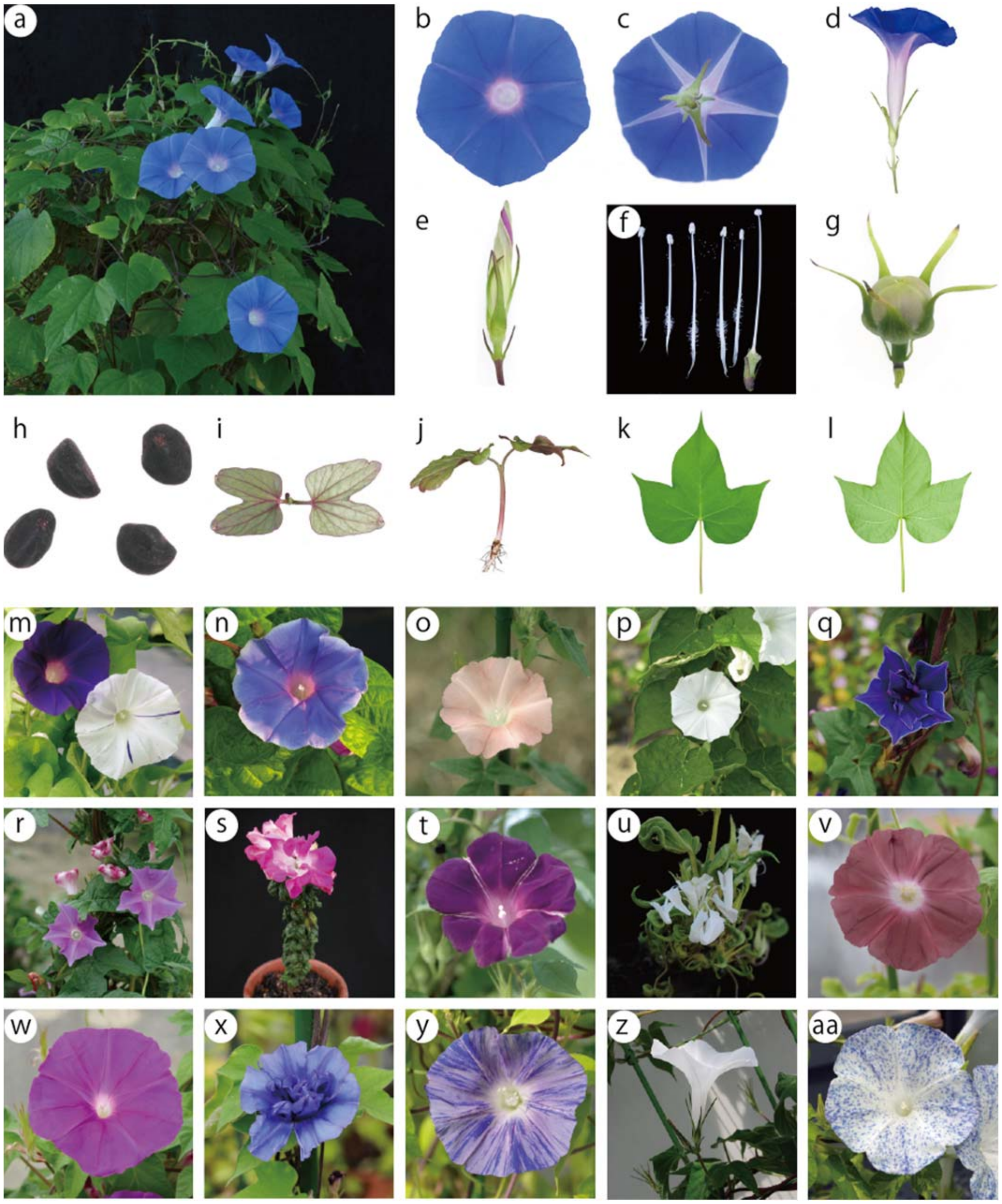

Supplementary Figure S1 | The Japanese morning glory. (a-l) The wild-type line Tokyo-kokei standard (TKS). **(a)** The individual used for whole genome sequencing. **(b)** Flower from the front. **(c)** Flower from the back. **(d)** Flower from the side. **(e)** Flower bud one day before flower opening. **(f)** Stamens (five on the left) and carpel

(farthest right). **(g)** Seed pod. **(h)** Seeds. **(i)** Seedling from above. **(j)** Side view of a seedling. **(k)** Leaf from the front. **(l)** Leaf from the back. **(m)** The Q1072 line carrying the recessive *a3-f* mutation that is the *Tpn1* insertion into the *DFR-B* gene for flower pigmentation. The mutant produces white flowers with pigmented spots or sectors (right) and sometimes produces fully pigmented flowers (left). The pigmentation patterns are caused by *Tpn1*-inducing somatic mutations and indicate that transposases TnpA and TnpD of *Tpn1* family elements are active. **(n-s)** Brassinosteroid-deficient mutants. **(n)** Q848 (*ct-1*). **(o)** Q853 (*ct-2*). **(p)** Q220 (*ct-w*). **(q)** Q708 (*s*). **(r)** Q721 (*s*). **(s)** Q837 (*ct-1*, *s*). **(t-aa)** The mutant lines carrying one of the recessive mutations that were mapped on the classic linkage map. The *cd*, *fe*, *dy*, *a3*, *mg*, *dp*, and *dk-2* mutations were assigned to classic LG1, LG2, LG3, LG4, LG5, LG6, and LG10, respectively. The recessive mutations of *c1* and *sp* were also assigned to LG3. In this study, LG3N with *dy* and LG3S containing *c1* and *sp* were found to correspond to different chromosomes (Supplementary Table S22). **(t)** Q557 (*cd*) showing partial transformation of floral petals into sepals. **(u)** Q459 (*fe*) showing alteration of organ polarity. **(v)** Q114 (*dy*) with dark-colored flowers. **(w)** AK62/Violet (*mg*) with reddish flowers. **(x)** Q426 (*dp*), producing double flowers. **(y)** Q531 (*dk-2*) with pale- and dull-colored flowers. **(z)** AK33 (*c1*), producing white flowers with red stems. **(aa)** AK30 (*sp*) showing speckled flowers. All *I. nil* lines are from the National BioResource Project (http://www.shigen.nig.ac.jp/asagao/).

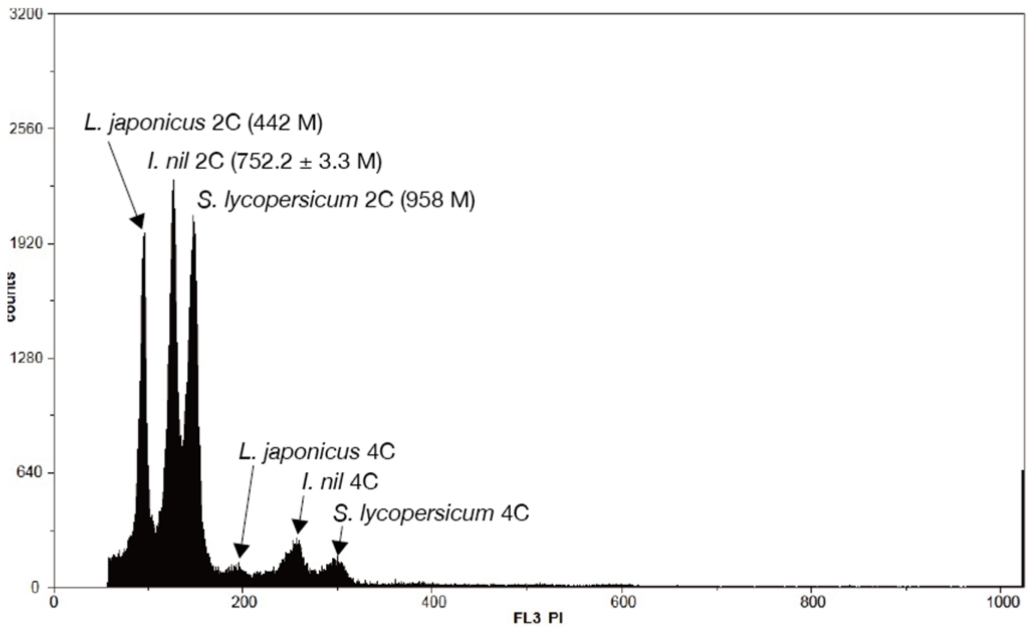

Supplementary Figure S2. A representative histogram of flow cytometric analysis for genome size estimation. Young leaves from the Tokyo-kokei standard of *I. nil* and the standard species were separately chopped using a razor blade for 1 min in an extraction buffer. *Lotus japonicus* Gifu and *Solanum lycopersicum* cv Micro-Tom were chosen as standard species[15,16]. The extraction buffer was PBS (pH 7.2) with 0.1 % (v/v) Triton X-100 and 400 µg/ml of RNase A (QIAGEN). After 15 min of incubation, the extracts were filtered using a 20-µm CellTrics disposable filter (Partec). Extracts from the three species were mixed at an appropriate ratio, and the mixtures were stained with 20 µl of propidium iodide solution (ICyt) per 1 ml of each mixture. All procedures above were done on ice. The three mixtures prepared from independent leaves of the species were analyzed by a flow cytometer, CyFlow SL, equipped with a 532-nm laser and FloMax software (Partec). The estimated genome size of *I. nil* is shown with a standard error (n=3).

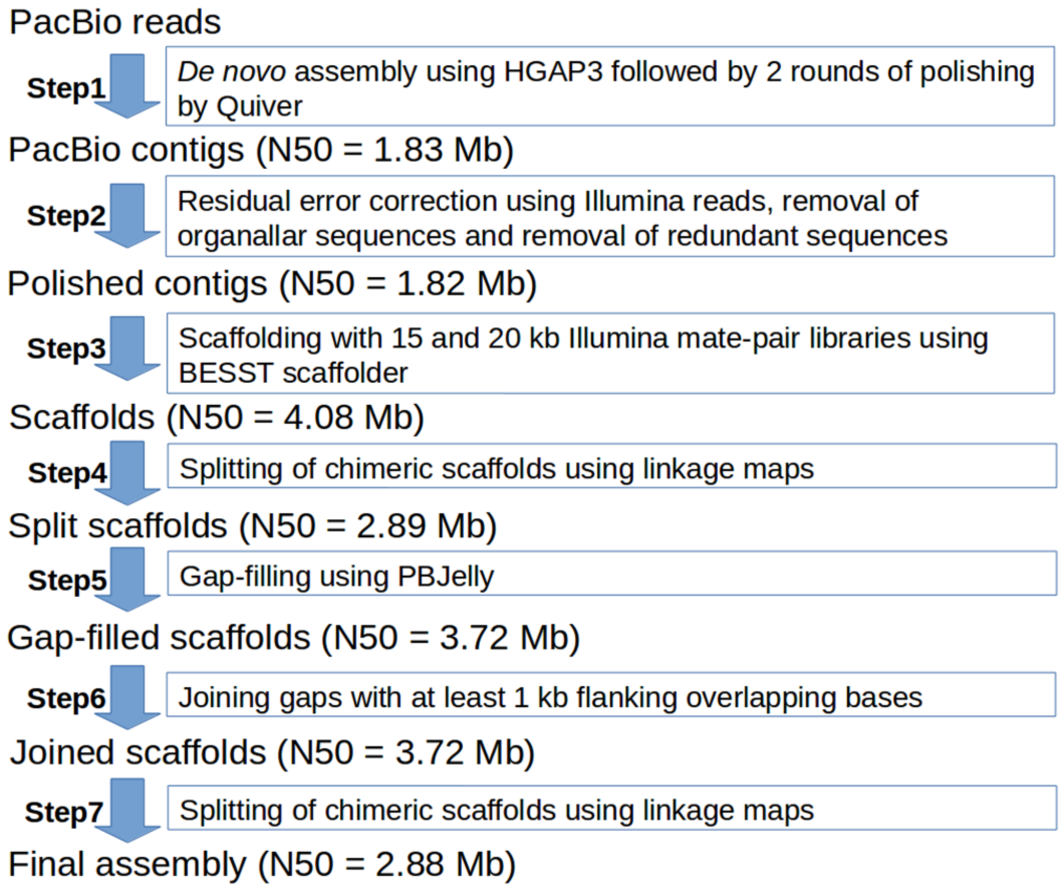

Supplementary Figure S3. Assembly work-flow. The assembly pipeline used for assembling the *I. nil* genome utilizing PacBio and Illumina sequence reads.

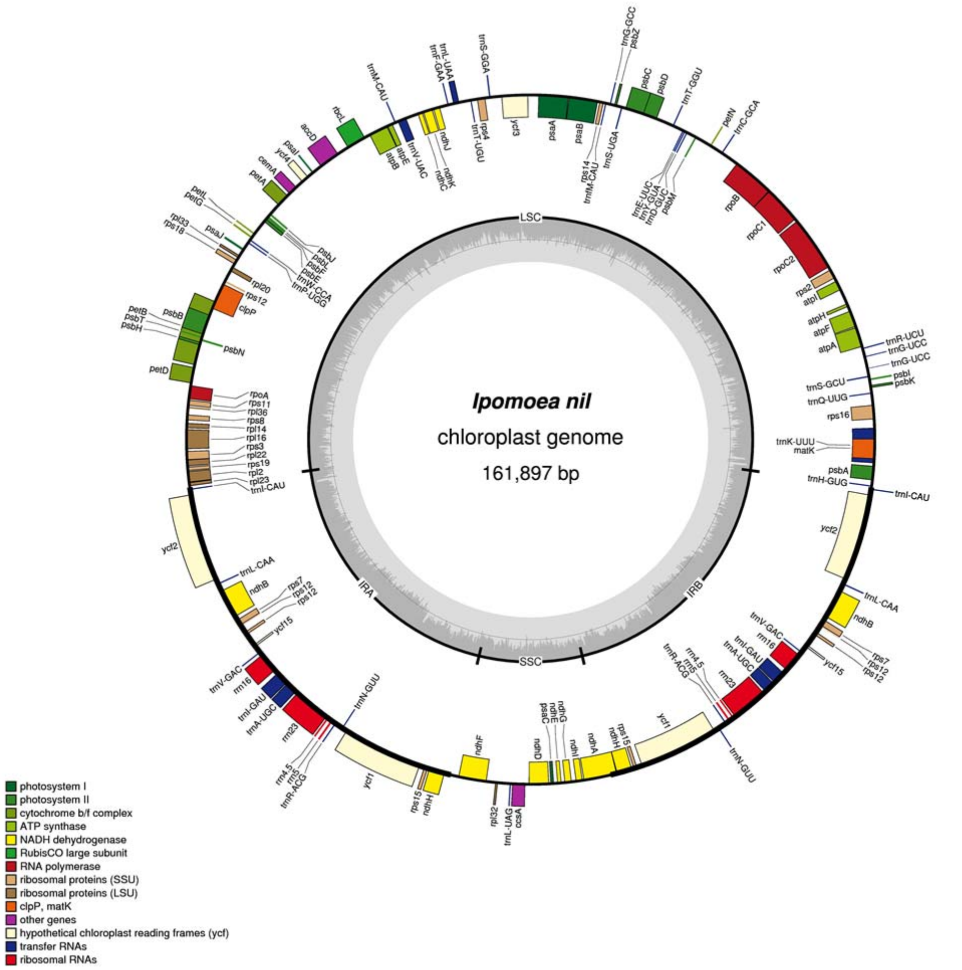

Supplementary Figure S4. The chloroplast genome of *I. nil*. The outer circle shows position of the genes including 88 protein coding, 38 tRNA, and 8 rRNA genes. The genes inside and outside the circle are transcribed in the clockwise and counterclockwise directions, respectively. The inner circle indicates the inverted repeats (IRA and IRB), small single-copy (SSC) and large single-copy (LSC) regions, and also shows the nucleotide content graph (G/C dark gray, A/T light gray).

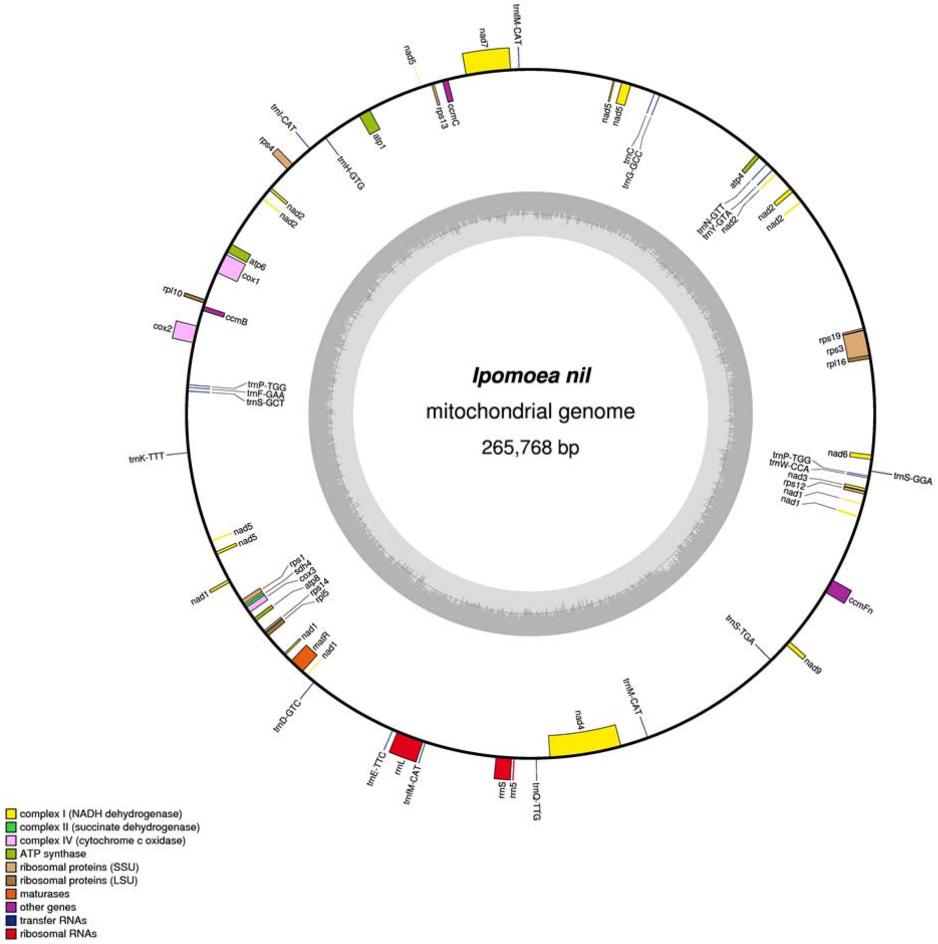

Supplementary Figure S5. The mitochondrial genome of *I. nil*. The outer circle shows position of the genes including 32 protein coding, 20 tRNA, and 3 rRNA genes. The genes inside and outside the circle are transcribed in the clockwise and counterclockwise directions, respectively. The pseudo genes for ccmFc and mttB found in the genome were removed from the map. The inner circle indicates the nucleotide content graph (G/C dark gray, A/T light gray).

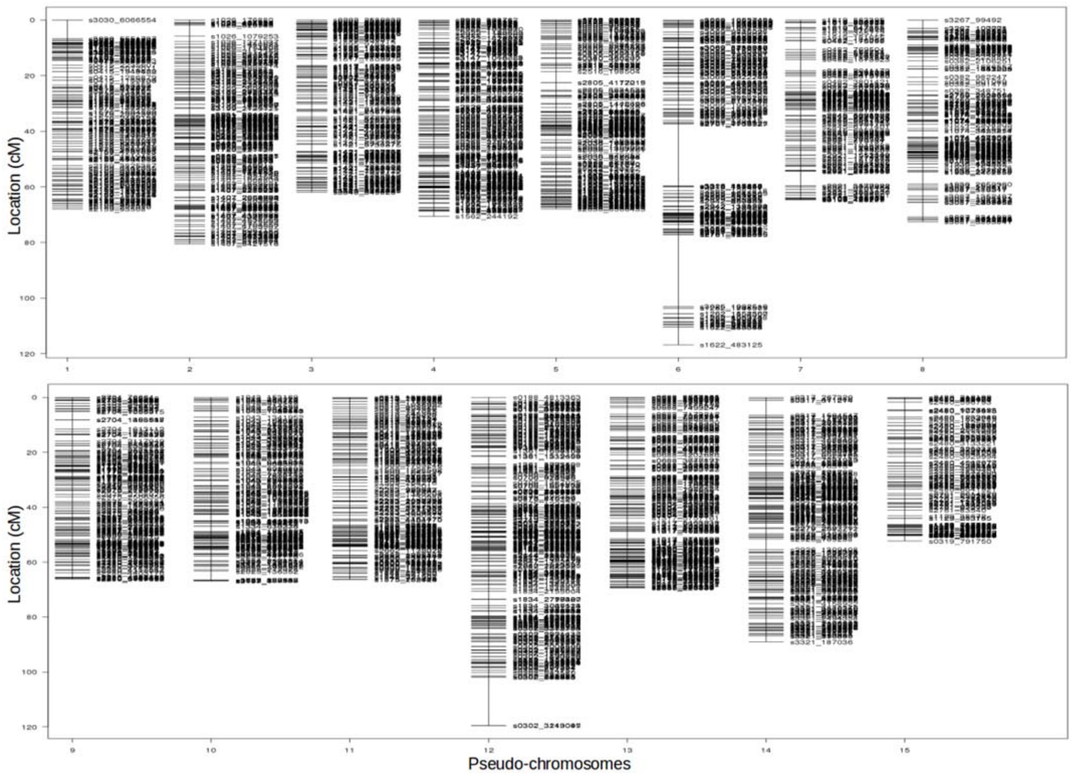

Supplementary Figure S6. Fifteen linkage maps. The markers correspond to scaffold names. For clarity of marker names, please refer to Supplementary Data 1.

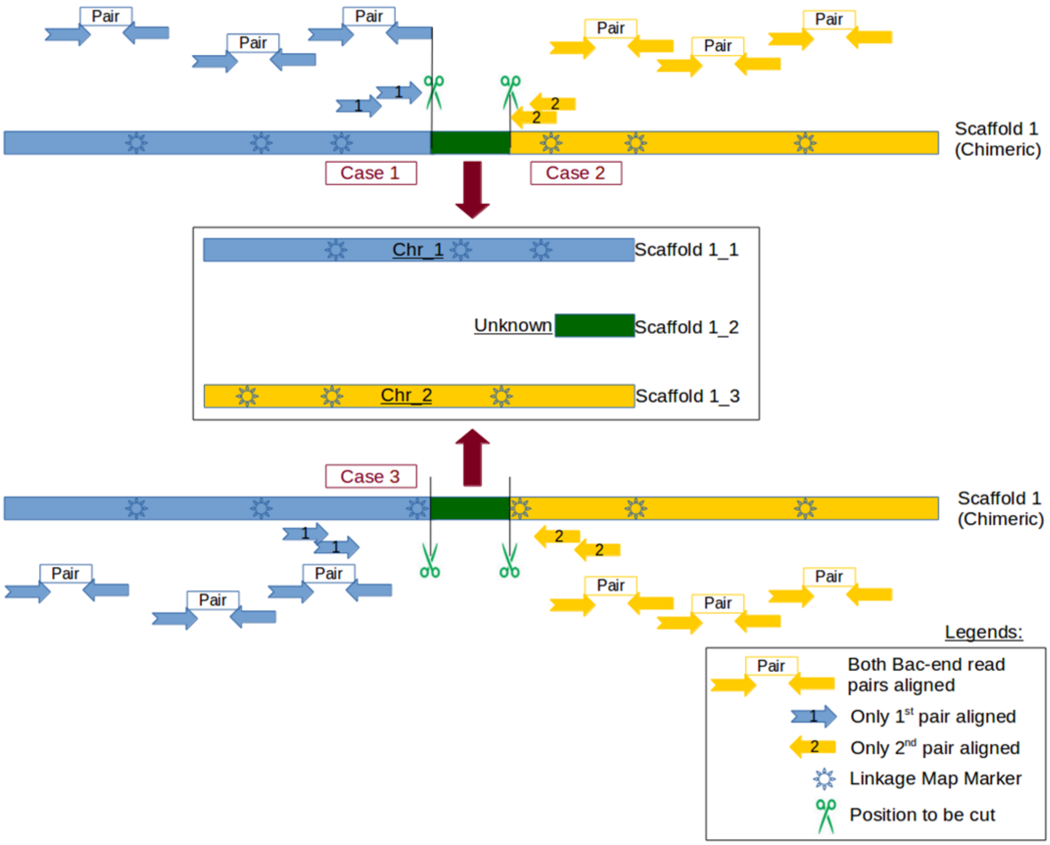

Supplementary Figure S7. Mis-assembly breakage process. Case 1 and 2 depicts breakage using BAC-end pair information. In case 1, the breakpoint is at the nearest complete BAC-end pair, and in case 2, the breakpoint is at the nearest BAC-end read, whose read-pair is in a different scaffold. Also, when there is not sufficient BAC-end read information, the SNP marker from the linkage maps was used as the breakpoint (Case 3). All cases were identified using disputes in linkage maps and were split into 3 separate scaffolds. The first and last scaffolds were assigned to corresponding chromosomes from the linkage map.

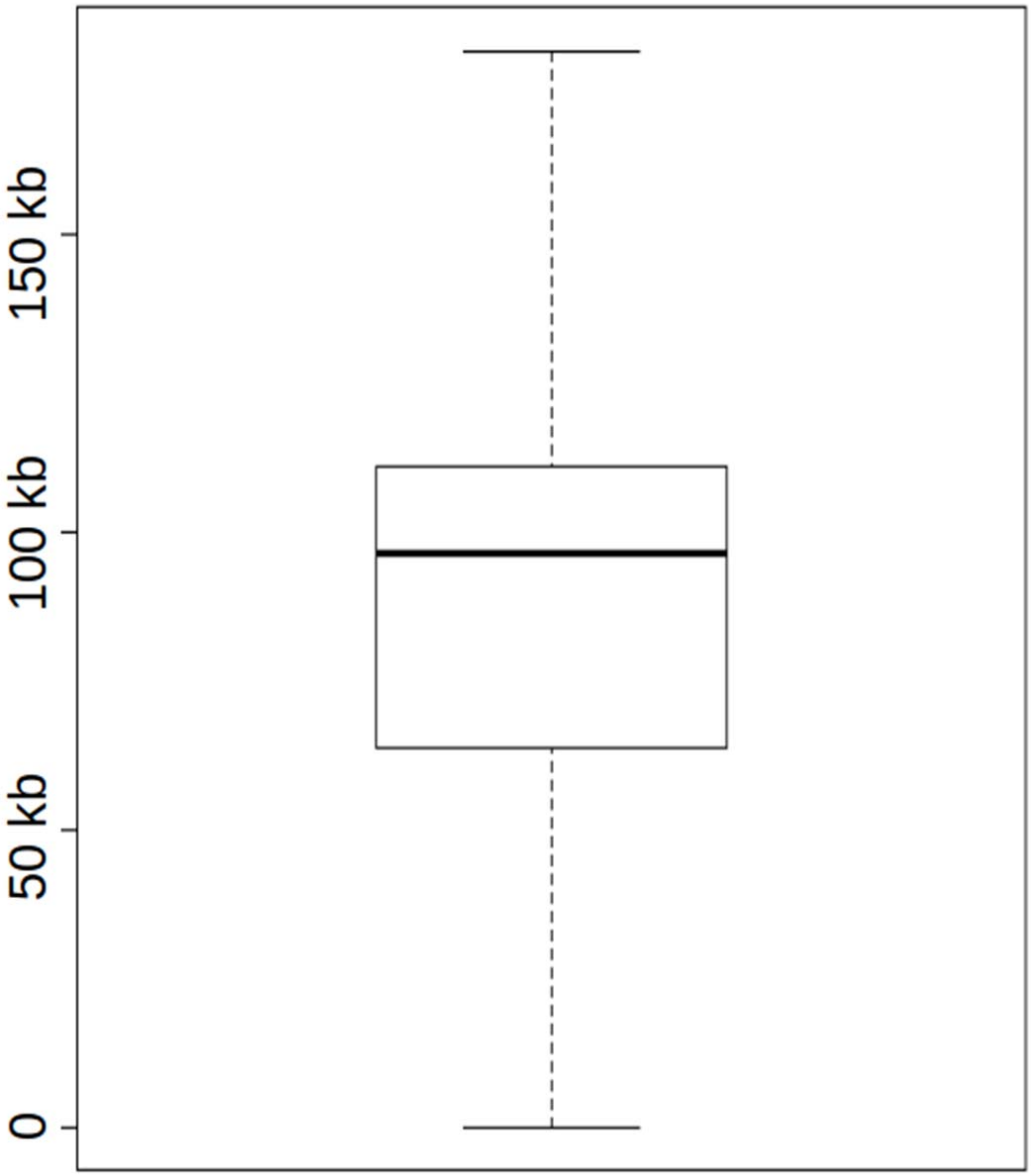

Supplementary Figure S8. Histogram of the observed BAC-end inserts. The BAC-end reads were aligned against the scaffolds, and the insert lengths between the pairs were calculated, and a histogram was plotted after removing outliers.

```
a                 TTATAAAATAGTGTAATACAATTCTCTTTGCAC-ATCTATACTATATATAAAAACAAAAT
c                 TTATAAAATAGTGTAATACAATTCCCTTTGCAC-ATCTATACTATATATAAAAACAAAAT
i                 TTTTAAAATAGTGTAATACAATTCCCTTTGTAC-ATCTATACTATATATAATAACAAAAT
h                 TTATAAAATAGTGTAATACAATTCCCTTTGCACAATCTATACTATATATAATAACAAAAT
e                 TTATAAAATAGTGTAATACAATTCCTATTGCA--ATCTATACTATATATAAAAACAAAAT
f                 TTATAAAATAGTGTAATACAATTCCTATTCTA--ATCTATACTATATATAATAACAAAAT
b                 TTATAAAATAGTGTAATACAATTCACTTTGCAC-ATCTATACTATATATAAAAACAAAAT
j                 TTTTAAAATAGTGTAATACAATTCCCTTTGTAC-ATCTTTACTATATATAATAATAAAAT
g                 TTTTAAAATAGTGTAATATAATTCCCTTTGTAC-ATCTATACTATATATAATAATAAAAT
d                 TTATAAAATA-TGTAATATAATTCAATTTGCAC-ATCTATACTATATATATAAACAAAAT
                  ** ******* *******.*****   ** .*  **** ***********  **.*****

a                 CCTGAAATTTTAACTTCCCGCCCAATTCCTAAAAAATCTCTCTTCCCAATAAAATATATT
c                 TCTGAAATTTTAACTTTCCGCCCAATTCCTAAAAAATCTCTCTTCCCAATAAAATATATT
i                 CCTGGAATTTTAACTTCCCGCCCAATTCCTAAAAAATTTCTCTTCCCAATAAAATATATT
h                 CCTGAAATTTTAACTTTCCGCCCAATTCCTAAAAAATCTCTCTTCCCAATAAAATATATT
e                 CCTGAAATTTTAACTTCCCGCCCAATTCCTAAAAAATCTCTCTTCCCAATAAAATATATT
f                 CCTAAAATTTTAACTTCCCGCCCAATTCCTAAAAAATCTCTCTTCCTCATAAAATATATT
b                 CCATAAATTTTAACTTTCCGCCCAATTCCTAAAAAATCTCTCTTCCCAATAAAATATATT
j                 CCTGGAATTTTAACTTCCCG-CCAATTCCTAAAAAATCTCTCTTCCCAATAAAATATATT
g                 CCTGGAATTTTAACTTACCGCTATATTCCTAAAAAATCTCTATTCCCAATAAAATATATT
d                 CCTAAAATTTTAACTTCCCGTCAAATTTATAAAATATCTCTCTTACCAATAAAATATATT
                  .*  .*********** ***  .   ***. ***** **.*** ** *. ************

a                 GATTTCTACTTTCCCATTCGTACAATAATTCTGGGTTAAAATTCTATTCTAAAA
c                 GATTTCTACTTTCCTATTCGTACAATAATTCTGGGTTAAAATTCTATTCTAAAA
i                 GATTTCTACTTTCCCATTCGTACAATAATTCTGGGTTAAAATTCTATTCTAAAA
h                 GATTTCTACTTTCCCATTCGTACAATAATTCTGGGTAAAAATTCTATTCTAAAA
e                 GATTTCTACTTTCCCATTCGTACAATAATTCTGGGTTAAAATTCTATTCTAATA
f                 GATTTCTACTTTCCCATTCGTACAATAATTCTGCGATAAAATTCTATTCTAATA
b                 GATTTCAACTATCCCATTCGTACAATAATTCTGTGTAAAAATTCTATTCTAAA-
j                 GATTTCTACTTTCCCATTCGTACAATAATTCTGTGTAAAAATTCTATTCTTAAA
g                 GATTTCTACTTTTCCATTCGTACAATAATTCTTGGTAAAAATTCTATTCTTAAA
d                 AATTTCTACTTTCCCATTAGTAAAATAGTT-TGAGTAAAAATTCTATTCTAAAA
                  .***** *** *.*.*** *** ****.** *  *  ************* *
```

Supplementary Figure S9. Putative consensus of the centromeric monomer repeats. MAFFT was used to construct multiple sequence alignment of ten random putative 173 bp centromeric monomer repeat sequences.

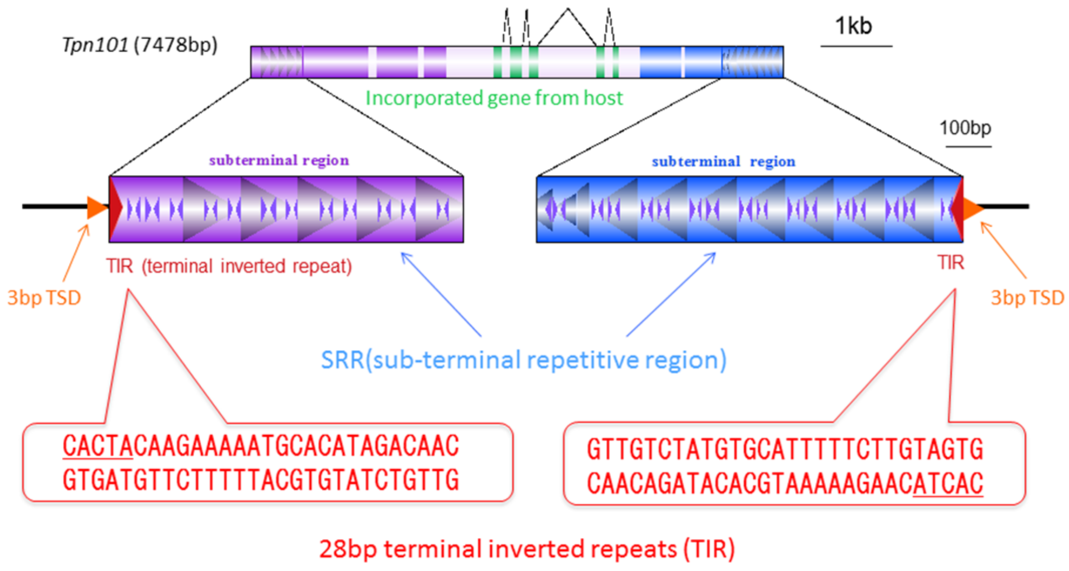

Supplementary Figure S10. Structure of a *Tpn1* family transposon. The first and last identical 28 bp represent Terminal Inverted Repeats (TIRs) across all the *Tpn1* transposons, flanked by typical 3-bp Transposon Site Duplications (TSDs). The TIRs are followed by sub-terminal repetitive regions (SRRs) and the region in-between can have incorporated genes from the host.

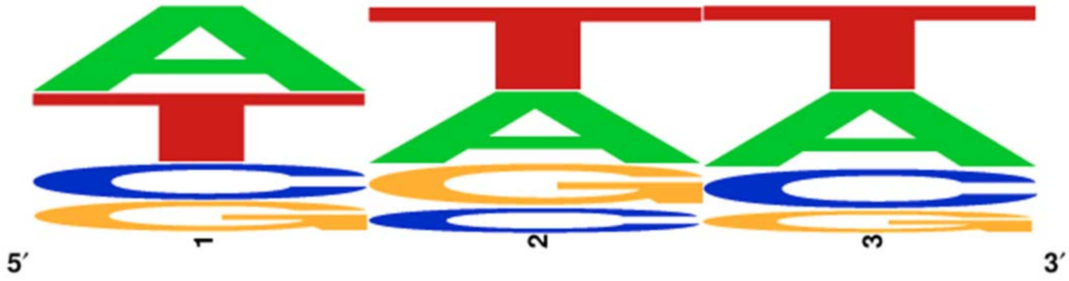

Supplementary Figure S11. Observed TSD motifs. The 3-bp TSDs of *Tpn1* transposons were used to create a motif pattern, which shows that TSDs are preferential to A and T nucleotides.

a

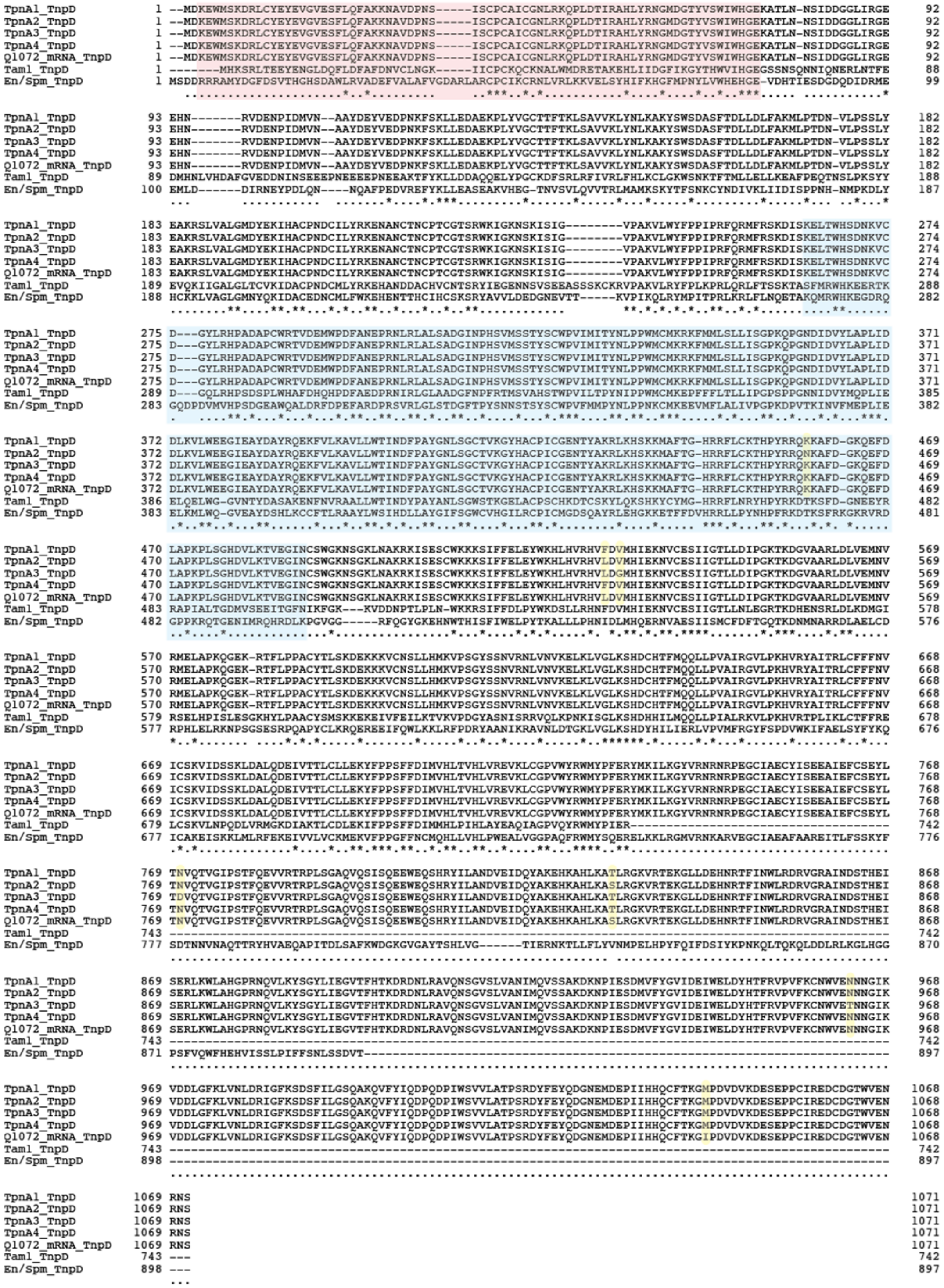

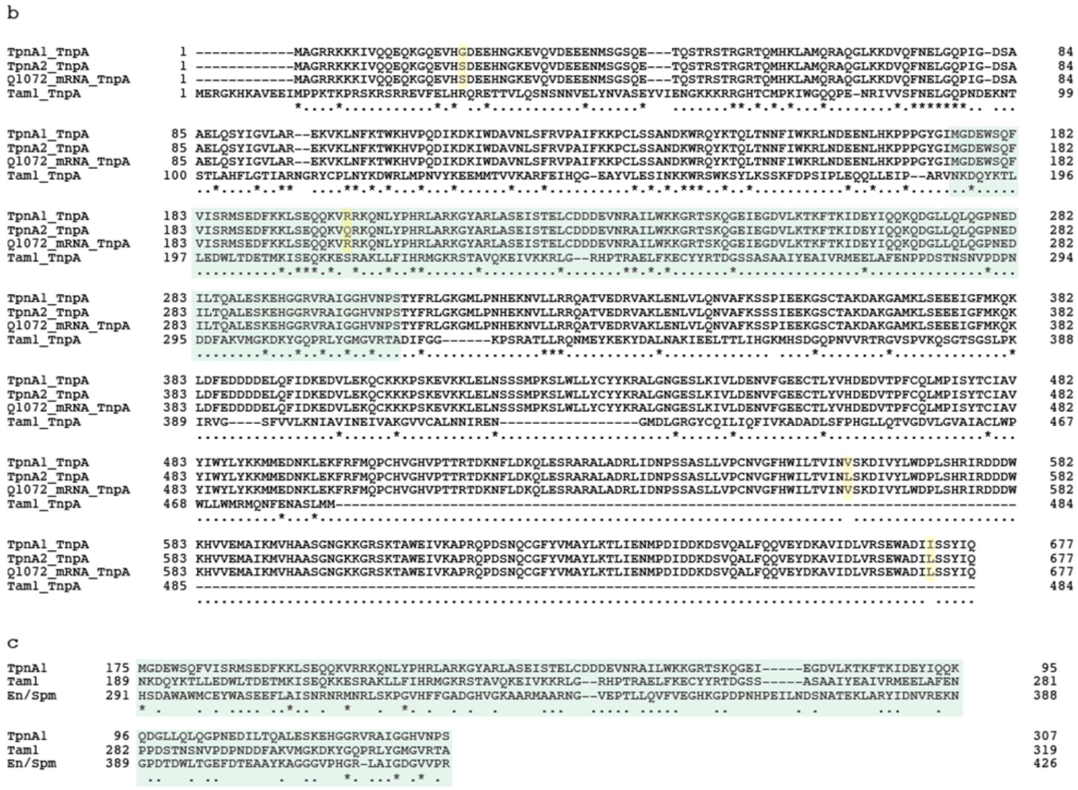

Supplementary Figure S12. Alignment of putative transposases. Amino acid sequences of TnpD transposases **(a)**, TnpA transposases **(b)**, and the conserved domain of TnpA transposases **(c)** from the *Tpn1* family elements, maize *En/Spm* (M25427), and snapdragon *Tam1* (X57297) were aligned. Yellow boxes show polymorphic amino acids among the *Tpn1* family elements. Bars show gaps. Asterisks and dots indicate amino acids conserved among all and parts of the aligned sequences respectively. Red, blue, and green shaded regions are conserved domains of transposase family tnp2 (pfam02992), transposase-associated domain (pfam13963), and plant transposase of *Ptta*/*En*/*Spm* family (pfam03004) respectively. Sequences were aligned using ClustalW[17] and conserved domains were searched using NCBI's Conserved Domain Database (CDD)[18].

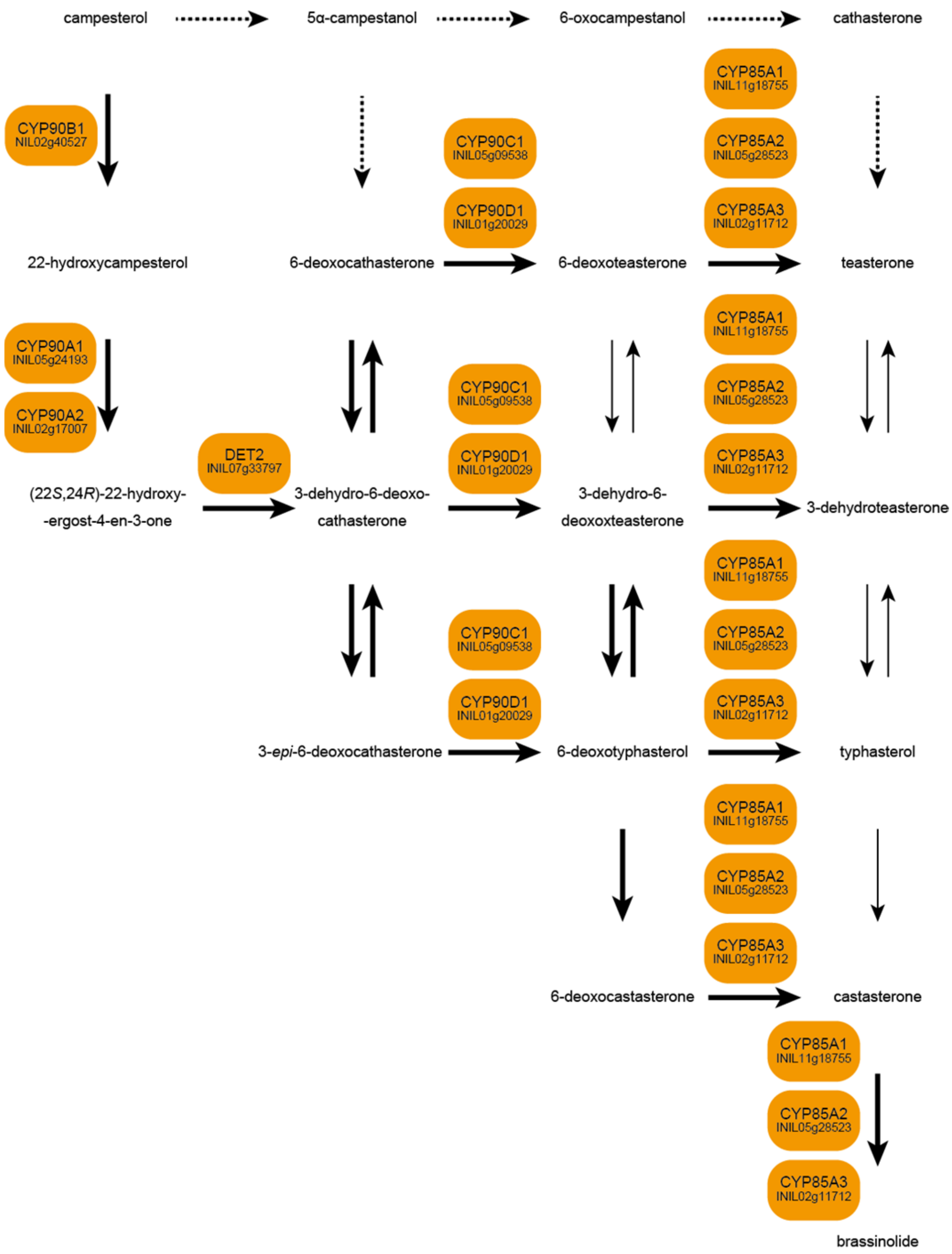

Supplementary Figure S13. A putative brassinosteroid biosynthesis pathway in *I. nil*. The enzyme names and *I. nil* gene IDs are represented in the colored rounded rectangles.

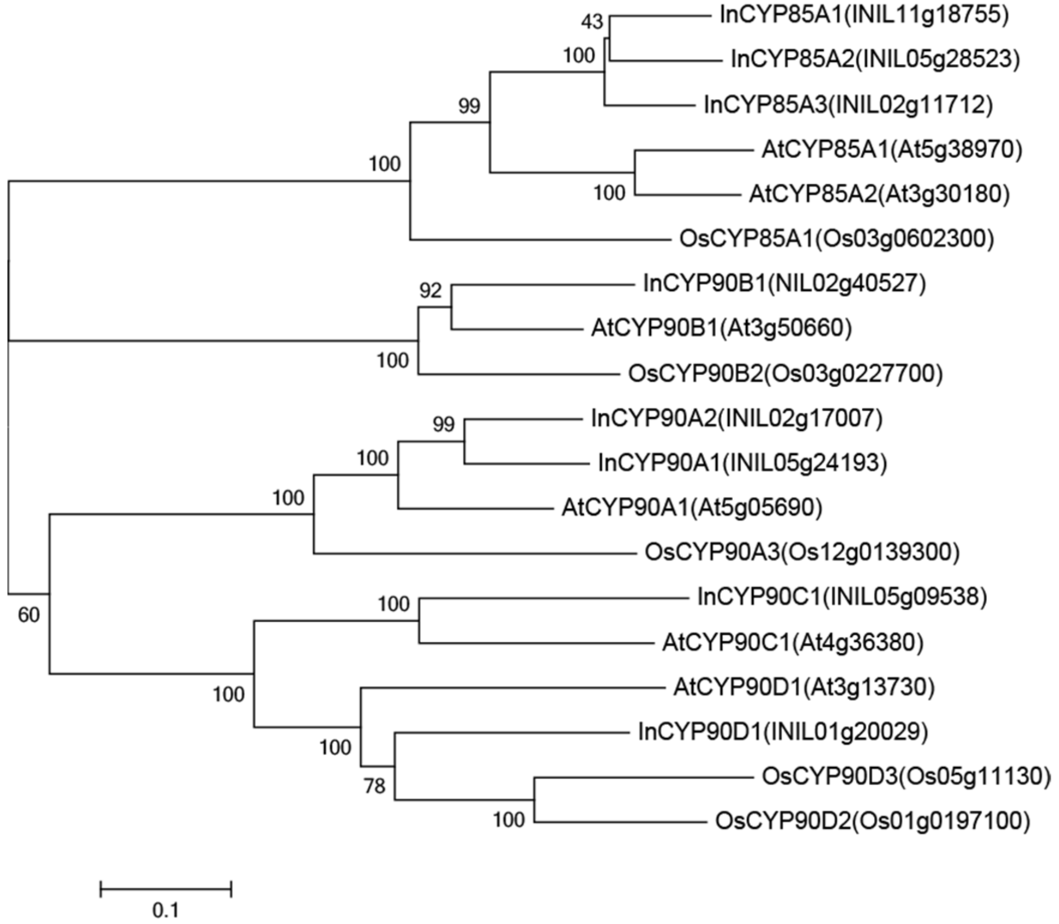

Supplementary Figure S14. A phylogenetic tree of P450 involving BR biosynthesis in *I. nil*, Arabidopsis, and rice. The tree was generated using the MEGA v5.2.2 program[19]. Amino acid sequences were aligned using ClustalW[17], and the tree was constructed by the neighbor-joining method. The bootstrap values out of 1,000 replicates are indicated at each branch, and the scale shows 0.1-amino acid substitutions per site. The *I. nil* gene IDs as well as Arabidopsis and rice loci are shown in parentheses.

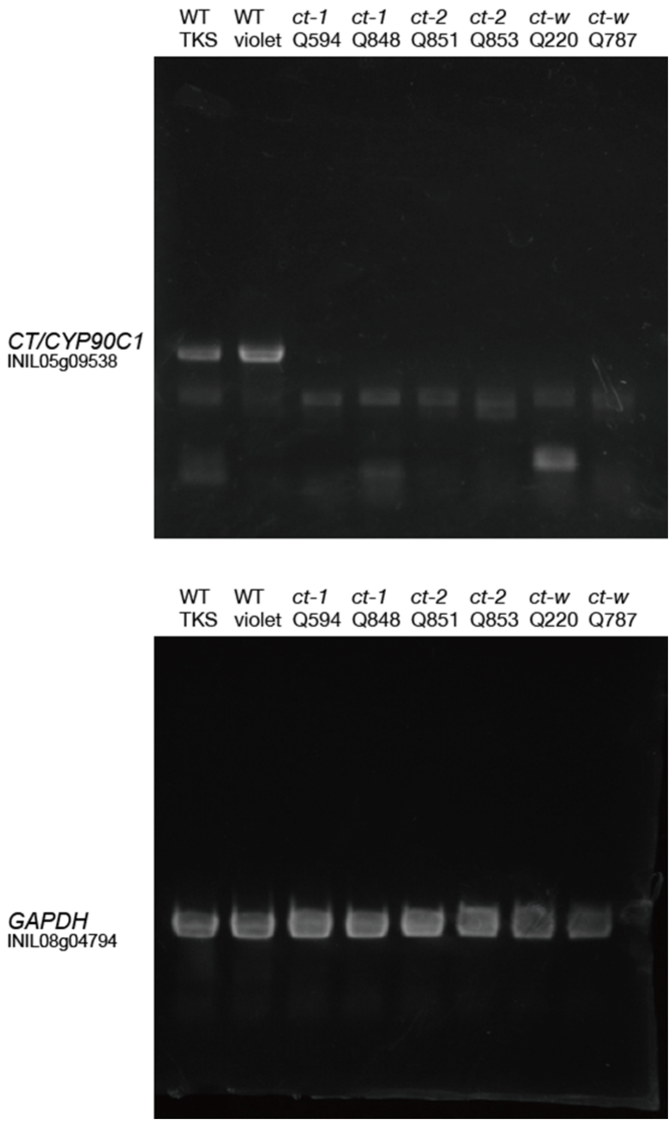

Supplementary Figure S15. Original images of ethidium bromide agarose gels shown in Figure 3c.

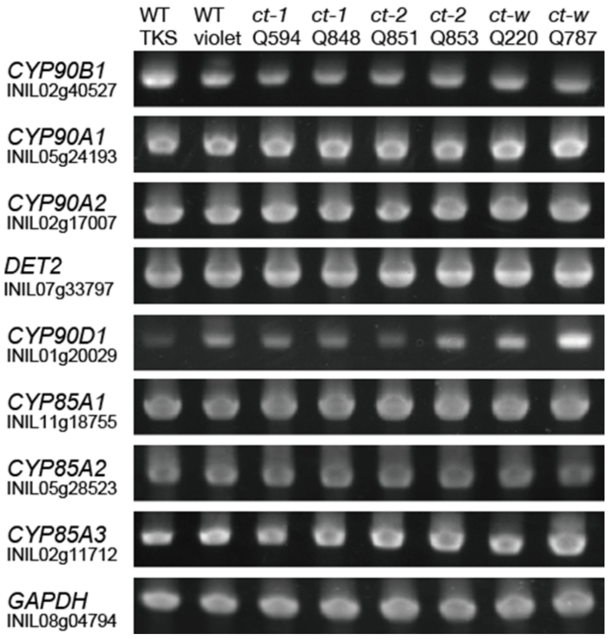

Supplementary Figure S16. Expression of the BR synthesis gene in the hypocotyls.

Expression was detected by reverse transcription PCR (RT-PCR). Representative gel images from three biological replicates were shown.

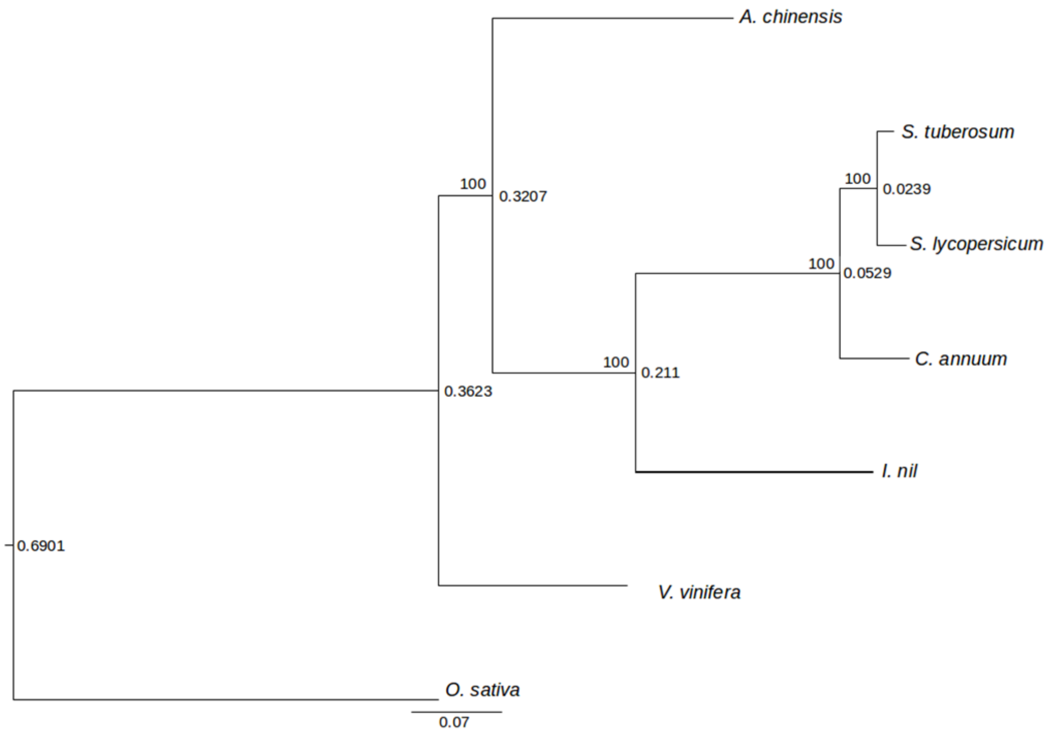

Supplementary Figure S17. Phylogenetic tree of the seven species used for comparative analysis. Maximum likelihood phylogenetic tree was generated using RaxML with a bootstrap value of 100. The scale bar (0.07) shows the number of substitutions per site.

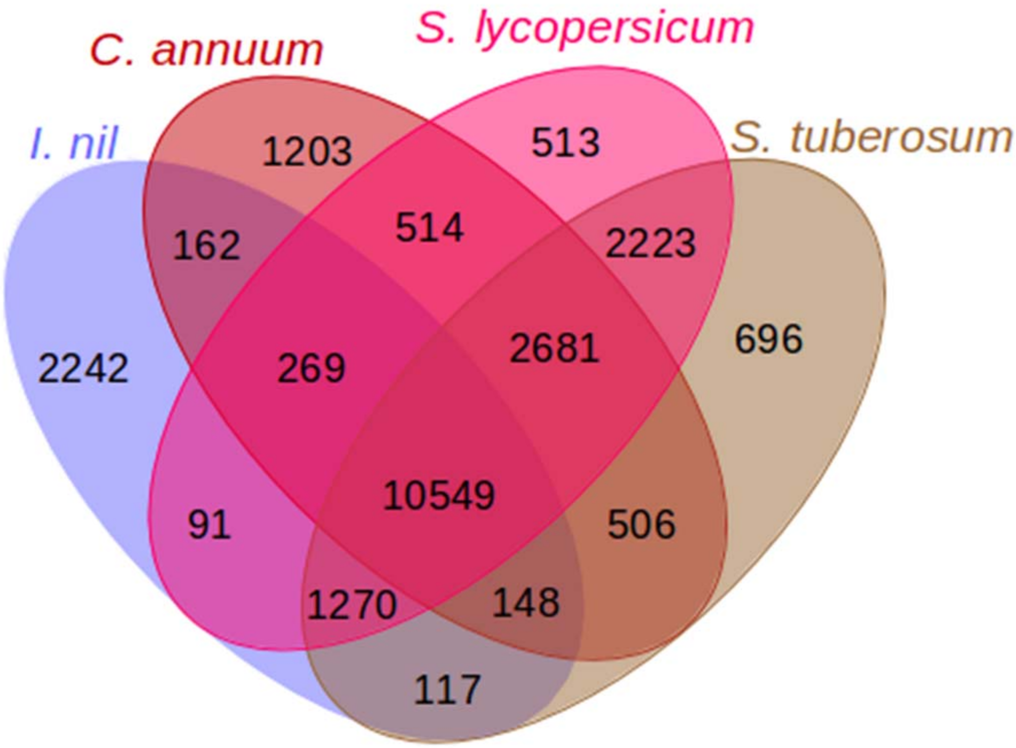

Supplementary Figure S18. Venn diagram depicting the gene family clustering of the Solanales.

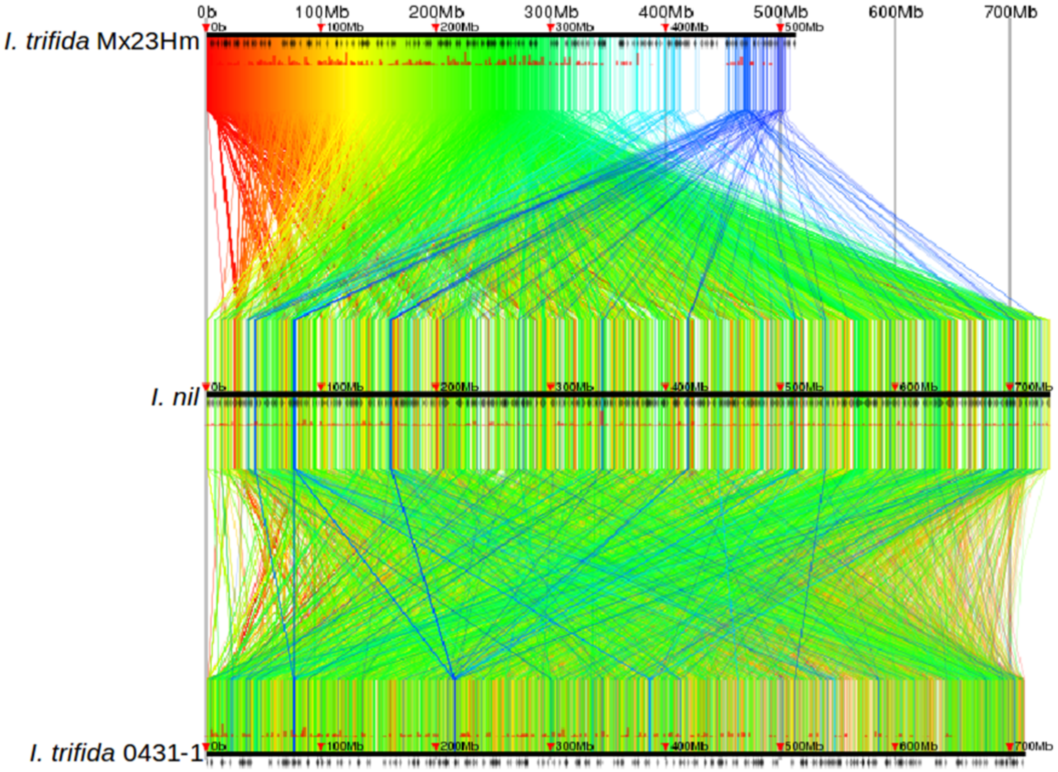

Supplementary Figure S19. Comparative alignment of *I. nil* and *I. trifida* genomes.

Whole genome alignments of the *I. nil* genome and the 2 lines of *I. trifida* generated using MURASAKI[20] with 100 bases as anchors for building the alignments.

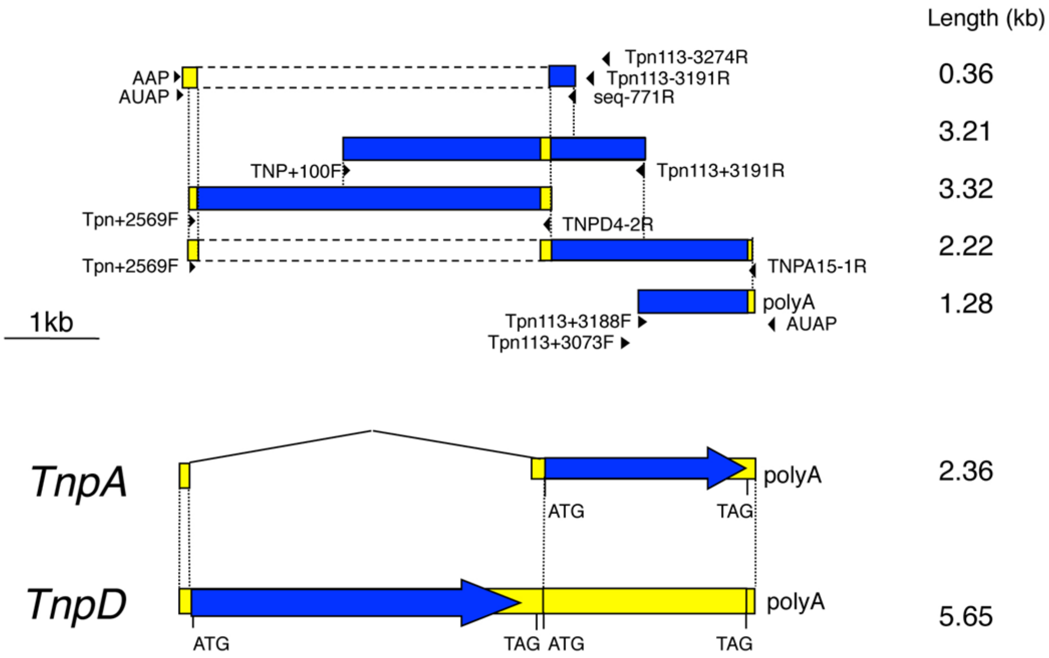

Supplementary Figure S20. A strategy for the isolation of *TnpA* and *TnpD* transcripts. Transcripts for TnpA and TnpD transposases were isolated from a mutable strain, Q1072. The yellow and blue boxes indicate untranslated regions and coding sequences respectively. The directions of the arrows show the orientations of the transposases. pA, ATG, and TAG indicate poly-adenines, start and stop codons respectively. Primer sequences are described in Supplementary Table S20.

Supplementary Tables

Supplementary Table S1. Statistics of raw Illumina reads

Strategy	Insert length	# of reads (in millions)	# of bases (in Gb)	Sequence coverage	Accession number
Paired end	300 bp	602	90	123 ×	DRR013917, DRR013918
Paired end	500 bp	652	98	133 ×	DRR013919, DRR013920
Mate pair	3 kb	563	85	115 ×	DRR013921, DRR013922
Mate pair	5 kb	544	82	111 ×	DRR013923, DRR013924
Mate pair	10 kb	584	88	119 ×	DRR013925, DRR013926
Mate pair	10 kb	505	76	103 ×	DRR048755
Mate pair	15 kb	495	74	101 ×	DRR048756
Mate pair	20 kb	494	74	101 ×	DRR048757

Supplementary Table S2. Comparison of the stepwise assemblies of PacBio data, with each step referring to the step from the assembly workflow (Supplementary Fig. S3)

	**Step 1**	**Step 2**	**Step 3**	**Step 4**	**Step 5**	**Step 6**	**Step 7**
					**Scaffolds**		
Sequences			3,367	3,495	3,345	3,345	3,416
Total Length of Sequences			734,061,355	734,055,648	735,418,791	734,768,050	734,803,190
Gap openings			624	602	544	461	449
Gap length			327,148	321,441	177,095	177,012	211,647
Longest sequence length			16,099,154	14,441,919	14,449,637	14,449,637	14,449,934
Shortest sequence length			638	638	638	638	638
Average sequence length			218,016.44	210,030.23	219,856.14	219,661.60	215,106.32
N50	NA		4,082,476	2,890,004	3,727,853	3,727,853	2,880,368
Sequences (>10 Mb)			7	2	6	6	2
Sequences (>1 Mb)			166	204	182	182	205
Sequences (>100 kb)			299	401	326	326	389
Sequences (>10 kb)			2,043	2,164	2,120	2,120	2,194
Sequences (>1 kb)			3,349	3,473	3,329	3,329	3,404
Sequences (>500 bp)			3,367	3,491	3,341	3,341	3,416
Sequences (>100 bp)			3,367	3,491	3,341	3,341	3,416
				**Contigs**			
Sequences	4,187	3,991	3,991	4,097	3,889	3,806	3,865
Total Length of Sequences	736,457,052	733,734,371	733,734,207	733,734,207	735,241,696	734,591,038	734,591,543
Longest sequence length	11,504,781	11,504,932	11,504,932	8,729,492	11,281,532	11,281,532	9,127,415
Shortest sequence length	638	638	638	638	638	638	638
Average sequence length	175,891.34	183,847.25	183,847.21	179,090.60	189,056.75	193,008.68	190,062.49
N50	1,830,236	1,825,684	1,825,684	1,584,472	1,918,312	2,087,487	1,873,359
Sequences (>10 Mb)	4	4	4	0	3	3	0
Sequences (>1 Mb)	191	191	191	200	189	195	205
Sequences (>100 kb)	649	649	649	724	620	574	625
Sequences (>10 kb)	2,773	2,648	2,648	2,746	2,650	2,567	2,629
Sequences (>1 kb)	4,169	3,973	3,973	4,075	3,873	3,790	3,853
Sequences (>500 bp)	4,187	3,991	3,991	4,093	3,885	3,802	3,865
Sequences (>100 bp)	4,187	3,991	3,991	4,093	3,885	3,802	3,865

Supplementary Table S3. Comparison of the Illumina and PacBio assemblies

	**PacBio Assembly**	**Illumina Assembly**
Sequences	3,416	2,262,957
Total length of sequences	734,803,190	1,106,449,450
Gap openings	449	132,545
Gap lengths	211,647	74,798,170
Longest sequence length	14,449,934	18,182,283
Shortest sequence length	638	128
Average sequence length	215,106.32	488.94
N50 (sequences >1 kb)	2,880,368	3,532,667
Sequences (>10 Mb)	2	3
Sequences (>1 Mb)	205	213
Sequences (>100 kb)	389	387
Sequences (>1 kb)	3,404	3,927
Sequences (>100 b)	3,416	2,262,957

Mis-assemblies were not resolved in the Illumina based assembly.

Supplementary Table S4. CEGMA analysis results

	#Proteins	%Completeness	#Total	Average	%Ortho
Complete	234	94.35	454	1.94	55.13
Group 1	62	93.94	100	1.61	41.94
Group 2	52	92.86	84	1.62	36.54
Group 3	57	93.44	126	2.21	64.91
Group 4	63	96.92	144	2.29	74.6
Partial	247	99.6	539	2.18	61.13
Group 1	66	100	116	1.76	45.45
Group 2	56	100	104	1.84	48.21
Group 3	61	100	151	2.48	68.85
Group 4	64	98.46	168	2.64	81.25

Proteins = number of 248 ultra-conserved CEGs present in genome

% Completeness = percentage of 248 ultra-conserved CEGs present

Total = total number of CEGs present including putative orthologs

Average = average number of orthologs per CEG

% Ortho = percentage of detected CEGs that have more than 1 ortholog

Supplementary Table S5. BUSCO analysis results

Complete Single-Copy BUSCOs	911 (95 %)
Complete Duplicated BUSCOs	186 (19 %)
Fragmented BUSCOs	16 (1.6 %)
Missing BUSCOs	29 (3 %)
Total BUSCO groups searched	956

Supplementary Table S6. Alignment statistics of RNAseq data

Sample	Total reads	Paired mapping rate	Concordant mapping rate
Leaf	10,555,280	96.66%	94.10%
Embryo	13,217,158	94.75%	96.90%
Flower	11,692,810	96.46%	94.00%
Stem	11,493,650	96.38%	93.00%
Root	11,601,760	96.36%	93.20%
Seed coat	11,111,334	96.73%	89.10%

Supplementary Table S7. Validation using entire BAC clone sequences

Statistics	1	2	3	4	5
**BLATScore**	94,487	100,460	126,029	106,895	125,057
**Mismatches**	0	0	0	0	1
**QgapOpen**	2	5	2	10	0
**QgapBases**	2	11	2	381	0
**TgapOpen**	7	6	6	5	2
**TgapBases**	15	8	9	8	8
**Strand**	+	-	+	-	-
**Q**	JMHiBa001I06	JMHiBa001L04	JMHiBa010C11	JMHiBa037J13	JMHiBa038C09
**Q Length**	94,489	100,471	126,031	107,276	125,058
**Q Start**	1	1	1	1	1
**Q End**	94,489	100,471	126,031	107,276	125,058
**T**	BDFN01001932	BDFN01002922	BDFN01003321	BDFN01001622	BDFN01000202
**T Length**	4,383,442	1,727,344	7,168,875	720,308	2,880,368
**T Start**	3,161,503	1,506,449	636,499	101,555	75,108
**T End**	3,256,005	1,606,917	762,537	208,458	200,174

Q and T indicates query and target sequences respectively.

Supplementary Table S8. Telomere repeat statistics

Scaffold	Start	End	Length	Orientation	# Repeating units	Pseudo-chromosomes
BDFN01001407	6,507,520	6,510,126	2,607	3'	379.3	2
BDFN01001026	1	1,414	1,414	5'	203.3	2
BDFN01003340	373,876	377,001	3,126	3'	447.6	3
BDFN01002922	15	5,582	5,568	5'	797.9	4
BDFN01001719	538,273	539,293	1,021	5'	145.1	5
BDFN01003069	10,854,526	10,854,850	325	5'	47.1	6
BDFN01001622	1	2,780	2,780	3'	396	6
BDFN01003087	4,002,600	4,007,449	4,850	3'	707.4	8
BDFN01003267	1	3,440	3,440	5'	493	8
BDFN01002704	1	1,760	1,760	5'	254.6	9
BDFN01001043	1	5,813	5,813	5'	825.9	10
BDFN01000302	1	7,517	7,517	3'	1,085.4	12
BDFN01000688	8,724,735	8,729,492	4,758	5'	678	13
BDFN01000317	1	2,185	2,185	5'	314.7	14
BDFN01003321	1	1,885	1,885	3'	270	14
BDFN01002480	3	3,164	3,162	5'	454.9	15
BDFN01000242	1	8,918	8,918	-	1,260.3	-
BDFN01000287	1	1,736	1,736	-	248.9	-
BDFN01000409	4	2,254	2,251	-	335.6	-
BDFN01000413	1	3,520	3,520	-	504.4	-
BDFN01000448	1	11,350	11,350	-	1,597.9	-
BDFN01000658	3	4,407	4,405	-	633.1	-
BDFN01001077	3	6,554	6,552	-	937.1	-
BDFN01001434	1	2,617	2,617	-	376.4	-
BDFN01001461	1	7,302	7,302	-	1,053.6	-
BDFN01001704	1	32,455	32,455	-	4,613.9	-
BDFN01001887	1	4,704	4,704	-	679.9	-
BDFN01002452	1	3,490	3,490	-	493.7	-
BDFN01003094	3	7,951	7,949	-	1,148.3	-
BDFN01002227	14,878	16,227	1,350	-	199.3	-

Supplementary Table S9. rDNA statistics

Scaffold	Type	# Units
BDFN01000878	5S rDNA	10
BDFN01001902	5S rDNA	17
BDFN01001350	5S rDNA	26
BDFN01002927	5S rDNA	28
BDFN01001762	5S rDNA	31
BDFN01002297	5S rDNA	36
BDFN01000751	5S rDNA	39
BDFN01001488	5S rDNA	46
BDFN01001624	5S rDNA	46
BDFN01002378	5S rDNA	52
BDFN01002742	5S rDNA	56
BDFN01003242	5S rDNA	61
BDFN01003244	5S rDNA	62
BDFN01000391	5S rDNA	67
BDFN01003186	5S rDNA	69
BDFN01001947	5S rDNA	73
BDFN01003379	5S rDNA	73
BDFN01001885	5S rDNA	76
BDFN01002400	5S rDNA	82
BDFN01003084	5S rDNA	88
BDFN01002263	5S rDNA	174
BDFN01000032	NOR	2
BDFN01000065	NOR	2
BDFN01000140	NOR	2
BDFN01000149	NOR	2
BDFN01000194	NOR	2
BDFN01000206	NOR	2
BDFN01000396	NOR	2
BDFN01000476	NOR	2
BDFN01000477	NOR	2
BDFN01000486	NOR	2
BDFN01000636	NOR	2
BDFN01000693	NOR	2
BDFN01000770	NOR	2
BDFN01000983	NOR	2
BDFN01001025	NOR	2
BDFN01001076	NOR	2
BDFN01001398	NOR	2
BDFN01001459	NOR	2
BDFN01001531	NOR	2
BDFN01001545	NOR	2
BDFN01001609	NOR	2
BDFN01001675	NOR	2
BDFN01001766	NOR	2
BDFN01001889	NOR	2
BDFN01002029	NOR	2
BDFN01002172	NOR	2

BDFN01002178	NOR	2
BDFN01002597	NOR	2
BDFN01002934	NOR	2
BDFN01003076	NOR	2
BDFN01003229	NOR	2
BDFN01003233	NOR	2
BDFN01003259	NOR	2
BDFN01003310	NOR	2
BDFN01001688	NOR	3
BDFN01002002	NOR	3
BDFN01002009	NOR	3

Supplementary Table S10. Putative centromeric repeat statistics

Scaffold	ScaffoldStart	ScaffoldEnd	Chr	ChrStart	ChrEnd	Length
BDFN01002165	2,909,054	2,970,073	1	12,053,754	12,114,773	61,020
BDFN01002191	60,623	67,982	2	28,397,071	28,404,430	7,360
BDFN01000794	254,934	432,528	3	7,168,727	7,346,321	177,595
BDFN01000222	102,194	178,324	5	29,541,552	29,617,682	76,131
BDFN01001993	3,770,803	3,782,015	6	35,924,044	35,935,256	11,213
BDFN01002928	2,752,998	2,758,913	8	30,246,790	30,252,705	5,916
BDFN01003226	682,231	750,649	9	23,220,192	23,288,610	68,419
BDFN01003314	6,904,122	6,914,607	10	26,085,935	26,096,420	10,486
BDFN01002752	73,285	106,516	12	23,848,936	23,882,167	33,232
BDFN01003104	1,904,565	1,910,793	13	33,938,054	33,944,282	6,229
BDFN01002694	1,373,703	1,477,297	14	22,742,219	22,845,813	103,595
BDFN01000233	1	28,566	15	16,639,357	16,667,922	28,566

Chr represents pseudo-chromosomes.

Supplementary Table S11. Repeat statistics

Repeat Element	# Elements	Length	% of sequence
SINEs:	19,969	2,353,092 bp	0.32 %
LINEs:	34,813	26,762,658 bp	3.64 %
LINE1	19,519	18,890,912 bp	2.57 %
LINE2	1,639	283,754 bp	0.04 %
LTR elements:	162,828	159,326,417 bp	21.68 %
ERV_classI	390	288,209 bp	0.04 %
ERV_classII	344	78,469 bp	0.01 %
DNA elements:	157,639	41,114,566 bp	5.60 %
Unclassified:	638,987	164,554,486 bp	26.82 %
Total interspersed repeats:		426,666,146 bp	58.07 %
Small RNA:	14,163	18,956,859 bp	2.58 %
Satellites:	2,052	1,479,996 bp	0.20 %
Simple repeats:	334,984	19,883,956 bp	2.71 %
Low complexity:	31,512	1,652,290 bp	0.22 %
Total Repeats		465,044,229 bp	63.29 %

Supplementary Table S12. The proportion of LTR copia and LTR gypsy repeat elements in the 15 pseudo-chromosomes

	Copia		Gypsy	
**Pseudo-chromosome**	**Length**	**Percentage**	**Length**	**Percentage**
1	5,801,120	13.53	6,890,412	16.07
2	4,958,985	11.51	5,992,448	13.91
3	4,994,172	12.87	5,895,457	15.19
4	6,777,445	13.67	7,408,401	14.94
5	5,042,172	11.90	6,205,709	14.64
6	6,527,109	13.26	7,944,017	16.14
7	3,395,198	10.74	4,220,562	13.35
8	5,313,761	12.79	6,001,260	14.44
9	6,326,179	14.95	6,679,331	15.78
10	5,912,286	13.40	6,219,745	14.10
11	4,695,077	12.01	5,094,635	13.03
12	9,057,553	13.68	9,449,217	14.27
13	7,190,683	14.73	7,329,827	15.01
14	7,522,699	12.54	8,105,536	13.52
15	4,188,809	13.07	4,975,673	15.52
Unassigned	7,197,387	11.41	7,866,454	12.47
Total	94,900,635	12.92	106,278,684	14.46

Supplementary Table S13. List of 3-bp TSDs in predicted *Tpn1* transposons

TSD	# Copies	TSD	# Copies
TTA	22	ATC	4
AAT	20	ATG	4
ATT	17	CGC	4
AAA	15	GCA	4
TTT	14	GGG	4
ATA	13	GTA	4
TAT	11	TGC	4
AGT	10	CTG	3
GTT	9	GAA	3
CAC	8	GCC	3
CAT	8	GCT	3
TGA	8	GTG	3
AAG	7	TAG	3
ACA	7	TGG	3
ACT	7	CCC	2
CTT	7	CGA	2
TAA	7	CGT	2
TTC	7	GAC	2
AGA	6	GGA	2
CTC	6	TCT	2
TCA	6	ACC	1
TGT	6	CCA	1
AAC	5	CCG	1
CAA	5	GAG	1
CTA	5	GGC	1
GAT	5	GGT	1
TAC	5	GTC	1
AGC	4	TCC	1
AGG	4	TTG	1

Supplementary Table S14. List of the *Tpn1* transposons inserted in the predicted genes

Scaffold	TpnStart	TpnEnd	Gene ID	Location of Tpn in the gene	Best hit to the predicted genes		
					Accession Number	Description	E Value
BDFN01000146	4167271	4171345	INIL07g00616	Within 5' UTR	XP_010451516	PREDICTED: uncharacterized protein LOC104733649	0.098
BDFN01000317	1396039	1396973	INIL14g03735	Within Intron	EYU25856	hypothetical protein MIMGU_mgv1a004842mg	2.00E-113
BDFN01000382	2241510	2248682	INIL08g04773	Within Intron	XP_015076614	PREDICTED: protein SIEVE ELEMENT OCCLUSION B-like	0
BDFN01000684	564240	571930	INIL12g07315	Within Intron	|XP_016460392	PREDICTED: apyrase-like	0
BDFN01000761	1642180	1645470	INIL11g08821	Start of 5' UTR	XP_012075563	PREDICTED: uncharacterized protein LOC105636820	3.00E-09
BDFN01000813	998831	1003004	INIL11g09944	Within Intron	XP_009763476	PREDICTED: NEP1-interacting protein-like 1	2.00E-110
BDFN01000900	89223	95490	INIL11g10743	Within Intron	XP_002280746	PREDICTED: two-component response regulator ARR17 isoform X1	4.00E-24
BDFN01001026	581352	587956	INIL02g11577	Within Intron	XP_015879219	PREDICTED: metalloendoproteinase 3-MMP-like	7.00E-75
BDFN01001074	3432916	3439614	INIL08g13816	Within Intron	KOM31814	hypothetical protein LR48_Vigan01g137000	0.019
BDFN01001149	2211938	2227500	INIL06g14602	Within Intron	YP_007476106	hypothetical chloroplast RF19	0.034
BDFN01001179	2105495	2112352	INIL12g14752	Within Intron	KZV56143	hypothetical protein F511_28809	4.00E-12
BDFN01001310	1663686	1667705	INIL06g16228	Within Intron	KZM95221	hypothetical protein DCAR_018463	3.00E-05
BDFN01001566	175717	188666	INIL00g19501	Start of 5' UTR	OAE18503	hypothetical protein AXG93_163s1360	0.14
BDFN01001612	286033	292485	INIL04g20363	Within Intron	KYP49349	Retrovirus-related Pol polyprotein from transposon 17.6	2.00E-38
BDFN01001735	7087	13506	INIL14g21144	Within Intron	BAF64710	putative transposase	2.00E-54
BDFN01001834	5102907	5109996	INIL12g22234	Within Intron	XP_010262527	PREDICTED: oxysterol-binding protein-related protein 3A-like isoform X2	2.00E-80
BDFN01001940	1164961	1172051	INIL12g23290	Within Intron	XP_016562472	PREDICTED: exocyst complex component EXO70A1-like	2.00E-07
BDFN01002387	1026555	1030496	INIL13g27102	Within Intron	XP_012464232	PREDICTED: carboxypeptidase Y	7.00E-41
BDFN01002403	1403792	1411332	INIL08g27285	Within Intron	XP_016494310	PREDICTED: topless-related protein 3-like isoform X2	6.00E-128
BDFN01002422	566388	569002	INIL15g27411	Within 5' UTR	XP_016436617	PREDICTED: uncharacterized protein LOC107762748 isoform X1	7.00E-53
BDFN01002494	182596	188978	INIL14g28304	Within Intron	XP_009108869	PREDICTED: transcriptional corepressor LEUNIG	1.5
BDFN01002653	2117625	2124193	INIL15g29342	Within 5' UTR	XP_015960841	PREDICTED: uncharacterized protein LOC107484813	0.022
BDFN01002653	2207114	2222242	INIL15g29346	Within 5' UTR	XP_013452680	hypothetical protein MTR_6g471360	5.00E-04
BDFN01002847	414256	421345	INIL00g33995	Within Intron	EEF41191	conserved hypothetical protein	7.00E-22
BDFN01002957	552638	559727	INIL09g35808	Within Intron	XP_009591617	PREDICTED: TMV resistance protein N-like	1.00E-73
BDFN01003047	597586	604379	INIL04g36913	Within Intron	XP_015056343	PREDICTED: vicianin hydrolase-like	0
BDFN01003170	2450756	2457359	INIL12g40104	Within Intron	CDP00659	unnamed protein product	4.00E-175
BDFN01003265	81415	86451	INIL03g41086	Within Intron	XP_016564068	PREDICTED: uncharacterized protein LOC107862882	1.00E-161
BDFN01003321	5576870	5584444	INIL14g41885	Within Intron	XP_015168855	PREDICTED: uncharacterized protein LOC102579241	2.00E-33

Supplementary Table S15. Statistics of the predicted gene models

Genes	42,783
Transcripts with complete ORF	44,916
Transcripts with 1 exon	7,868 (17.52 %)
Transcripts with 2 exons	7,624 (16.98 %)
Transcripts with 3 exons	6,220 (13.84 %)
Transcripts with 4 exons	4,750 (10.58 %)
Transcripts with 5 exons	3,748 (8.34 %)
Transcripts with 6 exons	2,821 (6.28 %)
Transcripts with 7 exons	2,281 (5.07 %)
Transcripts with 8 exons	1,752 (3.90 %)
Transcripts with 9 exons	1,527 (3.39 %)
Transcripts with 10 exons	1,177 (2.62 %)
Transcripts with 11 exons	957 (2.13 %)
Transcripts with 12 exons	705 (1.56 %)
Transcripts with 13 exons	670 (1.49 %)
Transcripts with 14 exons	540 (1.20 %)
Transcripts with 15 exons	412 (0.91 %)
Transcripts with >15 exons	1,864 (4.14 %)

Supplementary Table S16. *I. nil* lines and their *CONTRACTED* genes for brassinosteroid synthesis

Line No.	Line	Phenotype	Alleles	Reference
AK1	Tokyo Kokei Standard	*CT*	*CT*	[14]
AK2	Africa	*CT*	*CT*	[21]
AK4	Pekin Tendan	*CT*	*CT*	[21]
AK5	KK/ZSK-2	*CT*	*CT*	[22]
AK13	Shiranami	*CT*	*CT*	[23]
AK14	Akatsuki-no-yuki	*CT*	*CT*	[23]
AK16	Orihime	*CT*	*CT*	[23]
AK17	Tancho	*CT*	*CT*	[23]
AK30	Sagaminoyuki	*CT*	*CT*	[23]
AK31	Shirakumo	*CT*	*CT*	[23]
AK29	54Y	*CT*	*CT*	[24]
AK30	Hatsushimo	*CT*	*CT*	[24]
AK31	NS/Wca1	CT	CT	[25]
AK33	78WWc1	CT	CT	[25]
AK42	Hamanosora	*CT*	*CT*	[22]
AK43	Hamanokagayaki	*CT*	*CT*	[22]
AK52	Scarlet O'hara	*CT*	*CT*	[22]
AK55	Unzen	*CT*	*CT*	[26]
AK56	Aogakiyama	*CT*	*CT*	[27]
AK57	Akatsukinomine	*CT*	*CT*	[27]
AK58	Akatsukkinomurasaki	*CT*	*CT*	[27]
AK59	Akatsukinoumi	*CT*	*CT*	[27]
AK60	Akatsukinonami	*CT*	*CT*	[27]
AK62	Violet	*CT*	*CT*	[22]
Q220		*ct-w*	*ct-w*	this study
Q312		*ct*	*ct-2*	this study
Q578		*ct-w*	*ct-w*	this study
Q594		*ct*	*ct-1*	this study
Q606		*ct-w*	*ct-w*	this study
Q611		*ct-w*	*ct-w*	this study
Q629		*ct-w*	*ct-w*	this study
Q646		*ct-w*	*ct-w*	this study
Q787		*ct-w*	*ct-w*	this study
Q807		*ct*	*ct-2*	this study
Q811		*ct-w*	*ct-w*	this study
Q834		*ct*	*ct-2*	this study
Q837*		*ct*	*ct-2*	[28]
Q840		*ct*	*ct-1*	this study
Q848		*ct*	*ct-1*	this study
Q851		*ct*	*ct-2*	this study
Q853		*ct*	*ct-2*	this study
Q854		*ct*	*ct-2*	this study
Q857		*ct*	*ct-2*	this study

All *I. nil* lines listed here are from the National BioResource Project (http://www.shigen.nig.ac.jp/asagao/). *The Q837 line is heterozygous for the *star* mutation. The *ct* mutants had been classified into *ct* and *ct-w* by their characteristic phenotypes. The phenotypes of *ct* are slightly severer than those of *ct-w*.

Supplementary Table S17. Top 10 enriched GO terms in *I. nil* specific gene families

GO term	Description	p-value
GO:0051704	multi-organism process	1.90E-020
GO:0000003	reproduction	3.30E-019
GO:0009875	pollen-pistil interaction	3.30E-019
GO:0008037	cell recognition	3.30E-019
GO:0048544	recognition of pollen	3.30E-019
GO:0022414	reproductive process	3.30E-019
GO:0009856	pollination	3.30E-019
GO:0032501	multicellular organismal process	1.10E-016
GO:0007154	cell communication	2.00E-016
GO:0042545	cell wall modification	9.40E-010

Supplementary Table S18. Genome projects using PacBio sequence data for assembly improvement

Organism	Scaffold N50	Contig N50
PacBio data in later stages of assembly		
Conyza canadensis[29]	33.5 kb	20.7 kb
Saccharina japonica[30]	252 kb	58.8 kb
Belgica antarctica[31]	98.2 kb	-
Primula veris[32]	164 kb	9.4 kb
Nicrophorus vespilloides[33]	122.4 kb	102.1 kb
Lolium perenne[34]	70.1 kb	16.3 kb
Gossypium barbadense[35]	503 kb	72 kb
Dendrobium officinale[36]	25.1 kb	76.4 kb
Glanville fritillary[37]	119 kb	13 kb
Notothenia coriiceps[38]	129 kb	11.6 kb
Lingula anatine[39]	294 kb	55 kb
Thlaspi arvense[40]	140 kb	21 kb
PacBio data in contig assembly		
Gorilla gorilla	23 Mb	9.5 Mb
Lates calcarifer	1.19 Mb	1.06 Mb
Vigna angularis	-	3.0 Mb
Oropetium thomaeum	2.4 Mb	809.2 kb

Supplementary Table S19. Genome characterizations per pseudo-chromosome

Chromosome	Anchored Scaffolds	Length (Mb)	RAD markers	Length (cM)	Genes	*Tpn1* Elements
1	16	42.88	236	68.00	2,614	11
2	29	43.08	263	80.50	2,912	22
3	22	38.82	250	61.90	2,499	23
4	25	49.59	284	70.60	3,081	28
5	20	42.38	256	67.90	2,825	13
6	21	49.23	239	116.80	2,781	14
7	13	31.60	169	64.70	2,051	7
8	20	41.56	203	72.50	2,405	18
9	21	42.32	243	66.20	2,392	21
10	18	44.12	226	66.90	2,599	17
11	20	39.09	219	66.30	2,621	22
12	31	66.20	383	119.50	3,913	51
13	24	48.82	242	69.40	2,631	19
14	26	59.97	302	89.00	3,871	27
15	15	32.06	170	52.30	1,768	11
Total	321	671.72	3685	1132.50	40,963	304
Average	21.40	44.78	245.67	75.50	2,730.87	20.27

Supplementary Table S20. Primers and adapters used in this study

Gene, BAC clone	Primer, adapter	Sequence (5´ to 3´)	Usage
	Ara-1stBG	gAgAgAgAgAggATCCAACCCTggAgAgTTTTTTTTTTTTTTTTVN	Constructing the JMFF library
	Ara-Hairpin GN5	ggTTCTCgAgTCATCgCTgTTCCAgACAgCgATgACTCgAgAACCgNNNNN	Constructing the JMFF library
	1st strand	GAGAGAGAGAGGATCCTTTGGCCCTTATGGCCTACTTTTTTTTTTTTTTTVN	Constructing the JMSF library
	2nd strand	GAGAGAGAGAGGATCCTTTGGCCCTTATGGCCATCAC	Constructing the JMSF library
	TruSeq_MseI_NdeI_adaptor1	/5Phos/T*A*GAGATCGGAAGAGCACACGTCTGAACTCCAGTC*A*C	Preparing the RAD libraries
	TruSeq_MseI_NdeI_adaptor2	G*T*CAAGTTTCACAGCTCTTCCGATC*T*C	Preparing the RAD libraries
	TruSeq_BglII_adaptor1	G*A*TCGGAAGAGCTGTGCAGA*C*T	Preparing the RAD libraries
	TruSeq_BglII_adaptor2	A*A*TGATACGGCGACCACCGAGATCTACACTCTTTCCCTACACGACGCTCTT*C*C	Preparing the RAD libraries
TpnA1-4	AAP	GGCCACGCGTCACT-AGTACGGGIIGGGIIGGGIIG	cDNA cloning
TpnA1-4	AUAP	GGCCACGCGTCGACTAGTAC	cDNA cloning
TpnA1-4	AP	GGCCACGCGTCGACTAGTACTTTTTTTTTTTTTTTTT	cDNA cloning
TpnA1-4	seq-771R	GGTTGTCCTAGTTCATTGAATTGTA	cDNA cloning
TpnA1-4	TNP+100F	GGGAATGACATTGATGTTTATTTGG	cDNA cloning
TpnA1-4	TNPA15-1R	GTATTATTATGACTTGGATGATA	cDNA cloning
TpnA1-4	TNPD4-2R	AATTATACACTAGAAACTTGCTACG	cDNA cloning
TpnA1-4	Tpn+2569F	GTAATTGGCAGGTTGTTGCTGCTTC	cDNA cloning
TpnA1-4	Tpn113+3073F	GAATCTAAGGAGCATGGAGGTCGGG	cDNA cloning
TpnA1-4	Tpn113+3188F	GACGACAAGCAACAGTAGAAGACAG	cDNA cloning
TpnA1-4	Tpn113+3191R	CTCTGTCTTCTACTGTTGCTTGTC	cDNA cloning
TpnA1-4	Tpn113+3274R	CCTTTGCAGTACAACTCCCTTTTTC	cDNA cloning
CYP90C1	InCYP90-Fw1	TGAGAACGTATGTGGTTTTGGA	Amplify *InCYP90C1* coding region, and RT-PCR
CYP90C1	InCYP90-Rv1	ACAATCGTTTCCCTGGTTGA	Amplify *InCYP90C1* coding region, and RT-PCR

CYP90C1	InCYP90-Fw3	ACGGGAAGATACCATTGCTG	Amplify *InCYP90C1* promoter region
CYP90C1	InCYP90-Rv3	ATCCGGTTTTCTCGTCTCCT	Amplify *InCYP90C1* promoter region
CYP90C1	InCYP90-Fw4	GTTGAATTCCGGGGAAGAAT	Amplify *InCYP90C1* 3′ and downstream region
CYP90C1	InCYP90-Rv4	ACCCAACGTTTAGTGCATGA	Amplify *InCYP90C1* 3′ and downstream region
CYP90C1	InCYP90-Fw6	CACGTGTGACATGGTTTGGT	Amplify *Tpn15* insertion site of the *ct-1* and *ct-2* allele
CYP90C1	InCYP90-Rv6	TGGTTGGGGAAGTGAGTAGG	Amplify *Tpn15* insertion site of the *ct-1* and *ct-2* allele
CYP90C1	InCYP90-Fw11	TGATGCTCTGACTGCAAAAAGG	Amplify *Tpn16* insertion site of the *ct-w* allele
CYP90C1	InCYP90-Rv5	GGTTTCCCCAACAAGTGTGT	Amplify *Tpn16* insertion site of the *ct-w* allele
GAPDH	ipgap-forward	GCTTTAAGCCTCCGCCATGGG	RT-PCR
GAPDH	ipgap-reverse	ACGTTGGAAGCAATAAGCCCTTAAGCAG	RT-PCR
CYP90B1	INCYP90B1-F1	GGAGGAAGTCTGCAGTCAGCT	RT-PCR
CYP90B1	INCYP90B1-R1	CAATTCTGATCTGTCACTTCTGCT	RT-PCR
CYP90A1	INCYP90A1-F3	CCATGGAGTCATTGAATCTTCTT	RT-PCR
CYP90A1	INCYP90A1-R2	GGAAGTGTGGCCTCAAATGT	RT-PCR
CYP90A2	INCYP90A2-F1	GCTTTCTCCTTTAGCTGTTTTGGT	RT-PCR
CYP90A2	INCYP90A2-R1	TGCATCTACTAACACTCACTCTT	RT-PCR
DET2	INDET2-F1	AGAGATCCTTCCAGGTTGAAGA	RT-PCR
DET2	INDET2-R1	CAATCCAATTGCCATCACATTACTAC	RT-PCR
CYP90D1	INCYP90D1-F3	AACATCCCAATATGGACACCA	RT-PCR
CYP90D1	INCYP90D1-R1	GAGACGATACTGAAGAAAAGATGTGT	RT-PCR
CYP85A1	INCYP85A1-F1	GCCTTGAAAAGCGAAAACACCA	RT-PCR
CYP85A1	INCYP85A1-R1	GTGCTGTACATTTATTGAGTGATGG	RT-PCR
CYP85A2	INCYP85A2-F1	GTAGAGCTACTTAGCTTCATCA	RT-PCR
CYP85A2	INCYP85A2-R1	GTGGTATGTCCTTTTCTGTACA	RT-PCR

CYP85A3	INCYP85A3-F1	GAAGCTTCTTGGGAGGCCATT	RT-PCR
CYP85A3	INCYP85A3-R1	GTACATTCTCTGACTCATTGATG	RT-PCR
JMHiBa001I06	InCHI-B-F2	TGCAAATGTGGGTGGGATGATC	BAC library screening
JMHiBa001I06	InCHI-B-R1	ACTCCATAGGATCACCAAACTCTC	BAC library screening
JMHiBa001L04	C1865 LF1	GTGTAGGCTAGCTGGATAAGCCT	BAC library screening
JMHiBa001L04	C1865 R5	CCACCAGTGAGCCATAGCAAGAG	BAC library screening
JMHiBa010C11	XRN3-F1	CCTGGTGAAGGGGAACACAA	BAC library screening
JMHiBa010C11	XRN3-R3	CTCTGATGCAAGCGTGCTCT	BAC library screening
JMHiBa15I14	TpnA1-Fw1	TGCAGCCATGTCATGTAGGT	BAC library screening
JMHiBa15I14	TpnA1-Rv1	AATAGGCCGGTTGGATCATA	BAC library screening
JMHiBa037CJ13	XRN4-F1	ATGGAGTGGCTCCTAGAGCTA	BAC library screening
JMHiBa037CJ13	XRN4-R3	ATCTACGCCACGACAGTCA	BAC library screening
JMHiBa038C09	TNPA14-F1	GACTTTAATTGAGAACATGCCGGAC	BAC library screening
JMHiBa038C09	TNPA15-4R	TATTCCATAATCTCAAACATCTTTGTTC	BAC library screening

The asterisks indicate phosphorothioate bonds.

Supplementary Table S21. Tissues for the extraction of RNA for transcriptome analysis

Tissues	Sampling condition
Flowers	Tissues include sepals, petals, stamens, and carpels with short peduncles. Fully opened flowers, large flower buds (1–3 days before flower opening), and small flower buds (more than 4 days before flower opening) were separately collected in the evening.
Leaves	Various-sized leaves with short peduncles were mixed. Samplings were done at 4:30 on October 5, 2011 and at 14:30 on January 11, 2012.
Stems	Young stems, including the tips.
Seed coats	Seed coats on immature seeds in various developmental stages were mixed.
Embryos	Immature green embryos. Small embryos without bending cotyledon and large embryos with bending cotyledons were separately collected and subjected to RNA extraction.
Roots	Three-week-old roots cultured in vermiculite.

Flowers, leaves, stems, and seed coats are from a mature TKS plant using a whole genome shotgun sequence. Embryo and roots are from the progeny of the plant.

Supplementary Table S22. Comparison of the RAD-seq and the classical linkage map

Chromosome	LGs on classical map	RAD group	Marker gene	Scaffold	Reference or accession number
1	classic 1	RAD 1	*CONTORTED*	BDFN01003030	AB302068
2	classic 2	RAD 13	*FEATHERED*	BDFN01003169	[41]
3	classic 3N	RAD 10	*DUSKY*	BDFN01001421	[26]
4	classic 4	RAD 14	*MAGENTA*	BDFN01002848	[22]
5	classic 5	RAD 12	*A3*	BDFN01000805	[42]
6	classic 6	RAD 2	*DUPLICATE*	BDFN01003069	[43]
7	classic 7	RAD 6			
8	classic 8	RAD 3			
9	classic 9	RAD 4			
10	classic 10	RAD 11	*DUSKISH*	BDFN01001043	[44]
11	classic 3S	RAD 11	*C1*	BDFN01003238	[25]
11	classic 3S	RAD 15	*SPECKLED*	BDFN01001416	[45]
12		RAD 5			
13		RAD 8			
14		RAD 7			
15		RAD 9			

Supplementary Table S23. Starter materials for the EST analysis

Tissues	Sampling condition
Flowers, flower buds	Plant was grown in a greenhouse. Tissues include sepals, petals, stamens and carpels with short peduncles. Fully opened flowers and flower buds at 6 different stages were collected separately. The stages were 12 h and 36 h before flower opening, 30–40 mm, 20–30 mm, 10–20 mm, and less than 10 mm in length.
Seedlings	Plants were grown in a growth chamber, Biotron LH300 (Nippon Medical and Chemical Instruments) set to 28 °C. Aerial parts of the 8-day-old seedlings were collected. The light conditions were continuous light, 16 h light and 3 h dark after continuous light, and 10 h dark after continuous light.
Seed coats	Seed coats on immature seeds in various developmental stages were mixed. Plant was grown in a greenhouse.

Supplementary Table S24. EST libraries

Library name	Clones	Origin	End	ESTs	Accession number
JMFF	18,533	flowers, flower buds	5´	17,253	BJ553078–BJ566016<br>HY922663–HY926976
			3´	16,947	BJ566017–BJ578976<br>HY918676–HY922662
JMFN	9,011	flowers, flower buds	5´	9,011	HY926977–HY935987
JMFS	13,068	flowers, flower buds	5´	13,068	HY935988–HY949055
JMSF	19,476	Seedling	5´	18,627	CJ736987–CJ755610<br>HY949058–HY949060
			3'	17,714	CJ755611–CJ773322<br>HY949056–HY949057
JMCP	1,038	seed coat	5´	479	HY918197–HY918675
			3´	592	HY917605–HY918196
Total	61,126			93,691	

Supplementary Methods

DNA isolation

Genomic DNA for the shotgun sequence analysis was extracted from flower petals of young buds. Young buds were collected, frozen with liquid nitrogen, and stored at -80 °C until use. The 3-g sample was grounded to powder in liquid nitrogen with a mortar and pestle. The powder was dissolved quickly in 20 ml of Carlson lysis buffer preheated to 80 °C. The buffer was vigorously stirred with a hot magnetic stirrer and stir bar, and included 100 mM Tris-HCl (pH 9.5), 2 % CTAB (cetyltrimethylammonium bromide), 1.4 M NaCl, 1 % PEG6000, 20 mM EDTA (ethylenediaminetetraacetic acid), and 0.25 % β-mercaptoethanol. The extract was transferred to a 50-ml tube and incubated at 60 °C for 1 h on a water bath with shaking at 120 rpm. The sample was cooled to room temperature, and then 20 ml of chloroform/isoamyl alcohol (24:1) was added; it was mixed with a vortex mixer and rotated slowly with an overhead tube rotator. The extract was centrifuged at 8,000 g for 15 min at 4 °C. The aqueous phase was transferred into a fresh 50-ml tube, and 20 μl of 10 mg/ml RNase A (Nippon Gene) was added. After incubation for 30 min at 37 °C, 20 ml of Milli-Q was added to the sample, and the pH was adjusted to 7.0 with 25 % HCl. Genomic DNA was purified using a Genomic-tip 500/G (Qiagen) in accordance with the manufacturer's protocol and was resolved in 150 μl of Milli-Q.

To characterize the *CT* gene, genomic DNA was isolated from the leaves by either the NA-2000 or PI-480 (Kurabo) automated DNA isolation systems.

RNA isolation

Samples were collected, immediately frozen using liquid nitrogen, and stored at -80 °C until use. For RNA-seq analysis of the six tissues (Supplementary Table S21), RNA was extracted using a Get pureRNA Kit (Dojindo Molecular Technologies) and purified using an RNeasy Plant Mini Kit (Qiagen) in accordance with the manufacturer's protocols. To analyze *CT* expression and to isolate *TnpA* and *TnpD* transcripts, RNA was extracted using an RNeasy Plant Mini Kit (Qiagen).

Total RNA subjected to cDNA library constructions was isolated from tissues using a guanidinium isothiocyanate extraction buffer and purified by CsCl (cesium chloride) centrifugation. The tissues and stages of the sample are listed in Supplementary Table S23. Each 1-g sample was ground to powder in liquid nitrogen with a mortar and pestle, 10 ml of the extraction buffer was added, and it was then homogenized using an ultra disperser T25-S2 (IKA). The buffer contained 4 M guanidinium isothiocyanate, 0.1 M Tris-HCl, and 1 % β-mercaptoethanol, and the pH was adjusted to 7.0 using HCl. The samples were centrifuged at 12,000 g for 10 min at 4 °C. The supernatant was transferred to a new tube, to which a 0.025 volume of 1 M acetic acid and a 0.75 volume of ethanol were added; it was then chilled at -30 °C and centrifuged at 12,000 g for 20 min at 4 °C. The pellet was washed twice with 10 ml of 70 % EtOH and suspended in a sarkosyl buffer in a water bath set to 68 °C. The buffer contained 1 % (w/v) sodium lauroyl sarcosinate and 10 mM Tris-HCl; the pH was adjusted to 7.0 using HCl. The sample was then centrifuged at 8,000 g for 20 min at 4 °C. The supernatant was transferred to a new tube, and the volume was adjusted to 3 ml using the sarkosyl buffer before adding 3 g of CsCl. The sample was layered on a cushion of 1.25 ml of 5.7 M CsCl in the sarkosyl buffer and centrifuged at 21,000 g for 12 h at 4 °C using an RPS50-2 rotor (Beckman). The pellet was dissolved in 400 µl of Milli-Q; 40 µl of 3-M

sodium acetate (pH 5.2) and a 0.75 volume of ethanol were added, it was chilled at -30 °C, and was centrifuged at 20,000 g for 15 min at 4 °C. The pellet was then washed twice with 70 % EtOH, air-dried, and dissolved in Milli-Q.

cDNA library construction

The cDNA libraries made are listed in Supplementary Table S24. The JMFS, JMFN, and JMFF libraries were derived from mixtures of flowers and flower buds of the TKS line. JMSF and JMCP libraries were constructed from seedlings and seed coats, respectively, of TKS.

JMFS and JMFN stand for Japanese morning glory flower size-selected and normalized respectively. These libraries were constructed as described before[1]. Poly (A)+ RNA was purified using a Poly (A)+ RNA Quick mRNA Isolation Kit (Stratagene), and cDNAs were synthesized using a ZAP-cDNA Synthesis Kit (Stratagene) in accordance with the manufacturer's instructions. cDNA fragments with sequences 3 kb or longer were selected for the JMFS library and those ranging in lengths from 0.5 to 3 kb were normalized for the JMFN library, as described[1]. The size-selected and normalized cDNA fragments were separately cloned into the pBluescript II KS (+) (Stratagene) and transformed into *E. coli* DH10B. Each cDNA was cloned as an *Eco*RI and *Xho*I cassette.

JMFF and JMSF stand for Japanese morning glory flower full-length and Japanese morning glory seedling full-length respectively. These libraries house full-length-enriched cDNA. The cDNA was synthesized with trehalose-thremoactivated reverse transcriptase[2], and the CAP trapper method[3,4] was applied to select full-length cDNAs. For the JMFF library, Poly (A)+ RNA was purified using a Poly (A)+ RNA Quick

mRNA Isolation Kit (Stratagene). The primers of Ara-1stBG and Ara-Hairpin GN5 were used to synthesize the first and second strand cDNA respectively. The fragments were digested with *Xho*I and cloned into the *Sal*I site of the λFLC-I vector[5].

Construction of the JMSF library was ordered to Danaform. First and second strand cDNA was synthesized using 1st strand and 2nd strand primers respectively. One round of normalization was performed, as described[6], and they were then cloned into the λFLC-III vector[5]. The λ vector clones were subsequently converted into pFLC-I and pFLC-III phagemid derivatives by *in vivo* excision and transformed into phage resistant *E. coli* DH10B T1.

JMCP stand for Japanese morning glory seed coat PCR, and the JMCP library was constructed using the SMART cDNA Library Construction Kit (Clontech) in accordance with the manufacturer's protocol, with slight modification. After cDNA synthesis using long-distance PCR, the amplified cDNA fragments were cloned into pCR-XL-TOPO (Invitorogen) and transformed into *E. coli* TOP10.

EST analysis

Plasmid DNA was prepared from cDNA clones that were randomly chosen from the cDNA libraries. The 5′- and 3′-end sequences of the clones were determined using the ABI Prism 3100 Genetic Analyzer and ABI Prism 3700 Genetic Analyzer with BigDye version 3.1 chemistry (Applied Biosystems). The numbers of the clones analyzed as well as those of the obtained EST sequences are listed in Supplementary Table S24.

BAC library construction and end sequence

Construction of the BAC library was ordered to Clemson University Genomics Institute. Genomic DNA was isolated from pooled plants of TKS. *Bam*HI-digested DNA fragments were cloned into the pIndigoBAC536 vector and transformed into *E. coli* DH10B. A total of 27,648 clones were obtained. The average insert size was estimated to be approximately 100 kb. Of these clones, 5´- and 3´-ends of 23,424 clones were subjected to sequencing. BAC DNA was isolated from the clones and analyzed using an ABI Prism 3700 Genetic Analyzer with BigDye version 3.1 chemistry (Applied Biosystems). The BAC library was screened using a systematic PCR-based procedure, and six BAC clones—JMHiBa001I06, JMHiBa001L04, JMHiBa010C11, JMHiBa15I14, JMHiBa037J13, and JMHiBa038C09—were obtained. Of these, JMHiBa15I14 carries *TpnA1*, and the *TpnA1* sequence was analyzed using an ABI Prism 3100 Genetic Analyzer with BigDye version 3.1 chemistry (Applied Biosystems). The entire sequences of the remaining five BAC clones were sequenced using a shotgun sequencing procedure and were used for genome assembly validation.

Organellar genome sequence and annotation

BAC clones carrying the chloroplast and mitochondria genome fragments were selected by using the end sequences of the BAC clones. The clones are JMHiBa067I20, JMHiBa023J09, JMHiBa018N16, JMHiBa001E21 and JMHiBa032B12 for chloroplast genome and JMHiBa064B05, JMHiBa013H19, JMHiBa027O13, JMHiBa054N18 and JMHiBa056N18 for mitochondria genome. Shotgun libraries for the selected BAC clones were constructed and the shotgun clone sequences were determined using the ABI Prism 3700 Genetic Analyzer with BigDye version 3.1 chemistry (Applied Biosystems). The reads were analyzed with KB basecaller (KB1.2, Applied Biosystems)

for base calling, Phrap (v1.080812)[7] for assembling and Consed (v23.0)[8] for displaying and editing the assembled sequence. The assembled chloroplast and mitochondria genomes were annotated using DOGMA[9] and MITOFY[10] respectively. Initiation and stop codons as well as intron/exon boundaries were manually corrected. The published partial chloroplast genome of *I. nil* line REM459 (KF242487)[11] was used as a reference for manual correction. The organellar genome maps were generated using OrganellarGenomeDRAW[12,13].

Leaving out the partial and smaller overlapping contigs, the chloroplast and mitochondrial sequences were able to be completely reconstructed from just five and three sequences respectively in the PacBio based assembly. One of the three mitochondrial sequences (approximately 244 kb), which was merged as a chimeric mis-assembly with the end of a chromosomal contig, was separated manually prior to scaffolding.

Mis-assembly elimination at the contig level

When mis-assemblies, as predicted using linkage maps, occurred at the contig level rather than the scaffold level, the following method was followed to split the scaffolds. At the contig level, since there are no gap boundaries, it would be impossible to locate the exact junction point without a reference sequence. Hence, i) a larger chimeric region was identified using linkage maps; and ii) two breakpoints were induced at each side of the chimeric regions splitting the scaffold into 3 parts. The first part would map to one chromosome (linkage group), and the last part would map to a different chromosome, while the middle part would still remain chimeric; however, the length was narrowed to as short as possible. The following three cases were used to find the breakpoints in

chimeric regions: 1) the last base position, where both pairs of the BAC-end reads were concordantly mapped, 2) the last base position of a BAC-end pair where only one read was mapped near the scaffold, and 3) the base position after the SNP marker from the linkage maps (Supplementary Fig. S7). The breakpoints were then manually split to resolve mis-assembled contigs. Breakpoints were induced at positions, when there are at least 2 markers in a scaffold corresponding to two different pseudo-chromosomes from the linkage maps. The conservative strategy may lead to unnecessary contig breaking, with the tradeoff being shortening in contig lengths, however, the process ensures that there will be fewer mis-assemblies.

Isolation of the *TnpA* and *TnpD* transcripts

To isolate the transcripts derived from autonomous *Tpn1* family transposons, total RNA was extracted from the Q1072 strain, where *Tpn1* actively transposes. Primers were designed from a series of defective *Tpn1* family transposons[14] (Supplementary Fig. S20 and Supplementary Table S20). Sequence homology between defective *Tpn1* family transposons and other plant CACTA elements revealed that *Tpn113* contains a large part of the *TnpA* gene. Reverse transcription-PCR (RT-PCR) was performed using SuperScript First-Strand Synthesis System (Invitrogen) in accordance with the manufacturer's protocols. The 3.2-kb fragment was amplified with TNP+100F and Tpn113+3191R. TNPD4-2R was designed from this sequence. TNPD4-2R and Tpn+2569F were used to amplify the *TnpD* transcript. Using TNPA15-1R and Tpn+2569F, the *TnpA* transcript was obtained. 5´-RACE (rapid amplification of cDNA ends) was performed using 5´-RACE systems (Invitrogen) in accordance with the manufacturer's protocols. The reverse-transcription step was performed using

Tpn113+3274R. AAP (Abridge Anchor Primer) and Tpn113+3191R were used for the first amplification, and AUAP (Abridge Universal Anchor Primer) and seq-771R were used for the nested amplification. Tpn+2569F was designed from this sequence. 3´-RACE was performed using 3´-RACE systems (Invitrogen) in accordance with the manufacturer's protocols. Reverse transcription was used for the AP adapter primer. PCR was performed using Tpn113+31073F and AUAP pairs for the first PCR and Tpn113+3188F and AUAP pairs for the second PCR. TNPA15-1R was designed from this sequence. All cDNA sequences derived from putative autonomous *Tpn1* transposons were consistently compiled as full-length *TnpA* and *TnpD* transcripts.

Supplementary References

1. Asamizu, E., Nakamura, Y., Sato, S. & Tabata, S. A large scale analysis of cDNA in *Arabidopsis thaliana*: generation of 12,028 non-redundant expressed sequence tags from normalized and size-selected cDNA libraries. *DNA Res.* **7,** 175–180 (2000).
2. Carninci, P. *et al.* Thermostabilization and thermoactivation of thermolabile enzymes by trehalose and its application for the synthesis of full length cDNA. *Proc. Natl. Acad. Sci. U. S. A.* **95,** 520–524 (1998).
3. Carninci, P. *et al.* High-efficiency full-length cDNA cloning by biotinylated CAP trapper. *Genomics* **37,** 327–336 (1996).
4. Carninci, P. *et al.* High efficiency selection of full-length cDNA by improved biotinylated cap trapper. *DNA Res.* **4,** 61–66 (1997).
5. Carninci, P. *et al.* Balanced-size and long-size cloning of full-length, cap-trapped cDNAs into vectors of the novel lambda-FLC family allows enhanced gene discovery rate and functional analysis. *Genomics* **77,** 79–90 (2001).
6. Carninci, P. *et al.* Normalization and subtraction of cap-trapper-selected cDNAs to prepare full-length cDNA libraries for rapid discovery of new genes. *Genome Res.* **10,** 1617–1630 (2000).
7. Ewing, B. & Green, P. Base-calling of automated sequencer traces using *phred*. II. Error probabilities. *Genome Res.* **8,** 186–194 (1998).
8. Gordon, D., Abajian, C. & Green, P. Consed: A graphical tool for sequence finishing. *Genome Res.* **8,** 195–202 (1998).
9. Wyman, S. K., Jansen, R. K. & Boore, J. L. Automatic annotation of organellar genomes with DOGMA. *Bioinformatics* **20,** 3252–3255 (2004).

10. Alverson, A. J. *et al.* Insights into the evolution of mitochondrial genome size from complete sequences of *Citrullus lanatus* and *Cucurbita pepo* (Cucurbitaceae). *Mol. Biol. Evol.* **27,** 1436–1448 (2010).

11. Eserman, L. A., Tiley, G. P., Jarret, R. L., Leebens-Mack, J. H. & Miller, R. E. Phylogenetics and diversification of morning glories (tribe Ipomoeeae, Convolvulaceae) based on whole plastome sequences. *Am. J. Bot.* **101,** 92–103 (2014).

12. Lohse, M., Drechsel, O. & Bock, R. OrganellarGenomeDRAW (OGDRAW): A tool for the easy generation of high-quality custom graphical maps of plastid and mitochondrial genomes. *Curr. Genet.* **52,** 267–274 (2007).

13. Lohse, M., Drechsel, O., Kahlau, S. & Bock, R. OrganellarGenomeDRAW--a suite of tools for generating physical maps of plastid and mitochondrial genomes and visualizing expression data sets. *Nucleic Acids Res.* **41,** w575-w581 (2013).

14. Kawasaki, S. & Nitasaka, E. Characterization of *Tpn1* family in the Japanese morning glory: *En/Spm*-related transposable elements capturing host genes. *Plant Cell Physiol.* **45,** 933–944 (2004).

15. Doležel, J., Sgorbati, S. & Lucretti, S. Comparison of three DNA fluorochromes for flow cytometric estimation of nuclear DNA content in plants. *Physiol. Plant.* **85,** 625–631 (1992).

16. Ito, M. *et al.* Genome and chromosome dimensions of *Lotus japonicus*. *J. Plant Res.* **113,** 435–442 (2000).

17. Larkin, M. A. *et al.* Clustal W and Clustal X version 2.0. *Bioinformatics* **23,** 2947–2948 (2007).

18. Marchler-Bauer, A. *et al.* CDD: NCBI's conserved domain database. *Nucleic*

Acids Res. **43,** D222–D226 (2014).

19. Tamura, K. *et al.* MEGA5: molecular evolutionary genetics analysis using maximum likelihood, evolutionary distance, and maximum parsimony methods. *Mol. Biol. Evol.* **28,** 2731–2739 (2011).

20. Popendorf, K., Tsuyoshi, H., Osana, Y. & Sakakibara, Y. Murasaki: a fast, parallelizable algorithm to find anchors from multiple genomes. *PLoS One* **5,** e12651 (2010).

21. Ly, T. *et al.* Development of EST-SSR markers of *Ipomoea nil*. *Breed. Sci.* **62,** 99–104 (2012).

22. Hoshino, A. *et al.* Spontaneous mutations of the flavonoid 3'-hydroxylase gene conferring reddish flowers in the three morning glory species. *Plant Cell Physiol.* **44,** 990–1001 (2003).

23. Hoshino, A., Park, K. I. & Iida, S. Identification of *r* mutations conferring white flowers in the Japanese morning glory (*Ipomoea nil*). *J. Plant Res.* **122,** 215–222 (2009).

24. Abe, Y., Hohino, A. & Iida, S. Appearance of flower variegation in the mutable *speckled* line of the Japanese morning glory is controlled by two genetic elements. *Genes. Genet. Syst.* **72,** 57–62 (1997).

25. Morita, Y., Saitoh, M., Hoshino, A., Nitasaka, E. & Iida, S. Isolation of cDNAs for R2R3-MYB, bHLH and WDR transcriptional regulators and identification of *c* and *ca* mutations conferring white flowers in the Japanese morning glory. *Plant Cell Physiol.* **47,** 457–470 (2006).

26. Morita, Y. *et al.* Japanese morning glory *dusky* mutants displaying reddish-brown or purplish-gray flowers are deficient in a novel glycosylation enzyme for

anthocyanin biosynthesis, UDP-glucose:anthocyanidin 3-*O*-glucoside-2"-*O*-glucosyltransferase, due to 4-bp insertions. *Plant J.* **42,** 353–363 (2005).

27. Ting Sen, L., Norio, S., Masato, Y., Atsushi, S. & Toshio, H. Acylated peonidin glycosides in the violet-blue cultivars of *Pharbitis nil*. *Phytochemistry* **31,** 659–663 (1992).

28. Suzuki, Y. *et al.* A dwarf mutant strain of *Pharbitis nil*, Uzukobito (*kobito*), has defective brassinosteroid biosynthesis. *Plant J.* **36,** 401–410 (2003).

29. Peng, Y. *et al.* De novo genome assembly of the economically-important weed Horseweed using integrated data from multiple sequencing platforms. *Plant Physiol.* **166,** 1241–1254 (2014).

30. Ye, N. *et al. Saccharina* genomes provide novel insight into kelp biology. *Nat. Commun.* **6,** 6986 (2015).

31. Kelley, J. L. *et al.* Compact genome of the Antarctic midge is likely an adaptation to an extreme environment. *Nat. Commun.* **5,** 4611 (2014).

32. Nowak, M. D. *et al.* The draft genome of *Primula veris* yields insights into the molecular basis of heterostyly. *Genome Biol.* **16,** 12 (2015).

33. Cunningham, C. B. *et al.* The genome and methylome of a beetle with complex social behavior, *Nicrophorus vespilloides* (Coleoptera: Silphidae). *Genome Biol. Evol.* **7,** 3383–3396 (2015).

34. Byrne, S. L. *et al.* A synteny-based draft genome sequence of the forage grass *Lolium perenne*. *Plant J.* **84,** 816–826 (2015).

35. Liu, X. *et al*. *Gossypium barbadense* genome sequence provides insight into the evolution of extra-long staple fiber and specialized metabolites. *Sci. Rep.* **5,** 14139 (2015).

36. Yan, L. *et al.* The genome of *Dendrobium officinale* illuminates the biology of the important traditional Chinese orchid herb. *Mol. Plant* **8,** 922–934 (2015).

37. Ahola, V. *et al.* The Glanville fritillary genome retains an ancient karyotype and reveals selective chromosomal fusions in Lepidoptera. *Nat. Commun.* **5,** 4737 (2014).

38. Shin, S. *et al.* The genome sequence of the Antarctic bullhead notothen reveals evolutionary adaptations to a cold environment. *Genome Biol.* **15,** 468 (2014).

39. Luo, Y. J. *et al*. The *Lingula* genome provides insights into brachiopod evolution and the origin of phosphate biomineralization. *Nat. Commun.* **6,** 8301 (2015).

40. Dorn, K. M., Fankhauser, J. D., Wyse, D. L. & Marks, M. D. A draft genome of field pennycress (*Thlaspi arvense*) provides tools for the domestication of a new winter biofuel crop. *DNA Res.* **22,** 121–131 (2015).

41. Iwasaki, M. & Nitasaka, E. The *FEATHERED* gene is required for polarity establishment in lateral organs especially flowers of the Japanese morning glory (*Ipomoea nil*). *Plant Mol. Biol.* **62,** 913–925 (2006).

42. Inagaki, Y., Hisatomi, Y., Suzuki, T., Kasahara, K. & Iida, S. Isolation of a *Suppressor-mutator/Enhancer*-like transposable element, *Tpn1*, from Japanese morning glory bearing variegated flowers. *Plant Cell* **6,** 375–383 (1994).

43. Nitasaka, E. Insertion of an *En/Spm*-related transposable element into a floral homeotic gene *DUPLICATED* causes a double flower phenotype in the Japanese morning glory. *Plant J.* **36,** 522–531 (2003).

44. Morita, Y., Ishiguro, K., Tanaka, Y., Iida, S. & Hoshino, A. Spontaneous mutations of the UDP-glucose:flavonoid 3-*O*-glucosyltransferase gene confers pale- and dull-colored flowers in the Japanese and common morning glories.

Planta **242,** 575–587 (2015).

45. Hoshino, A., Johzuka-Hisatomi, Y. & Iida, S. Gene duplication and mobile genetic elements in the morning glories. *Gene* **265,** 1–10 (2001).
